# Genomic and virulence characterization of OXA-48-producing *Klebsiella pneumoniae* causing urinary tract infections and their susceptibility to lytic bacteriophages

**DOI:** 10.3389/fmicb.2026.1849761

**Published:** 2026-06-09

**Authors:** Sonia Rey, Sergio Silva-Bea, Carlos Davina-Nunez, Manuel Romero, Belén Fontán-Silva, Sonia Pérez, Ana Otero

**Affiliations:** 1 Microbiology and Parasitology Department, Complexo Hospitalario Universitario de Vigo (CHUVI), Servicio Galego de Saúde (SERGAS), Vigo, Spain; 2 Microbiology and Infectology Research Group, Galicia Sur Health Research Institute (IIS Galicia Sur), Vigo, Spain; 3 Department of Microbiology and Parasitology, Faculty of Biology, Universidad de Santiago de Compostela, Santiago de Compostela, Spain; 4 Aquatic One Health Research Center (ARCUS), Universidad de Santiago de Compostela, Santiago de Compostela, Spain

**Keywords:** bacteriophage, biofilm, carbapenemase, *Klebsiella pneumoniae*, MDR, OXA-48, urinary tract infection, UTI

## Abstract

**Introduction:**

*Klebsiella pneumoniae* (Kpn) is the second most frequent cause of urinary tract infections (UTIs) and represents a major concern in the context of antimicrobial resistance (AMR); nevertheless, the phenotypic and genomic traits that define uropathogenic Kpn strains are not clear.

**Methods:**

In this study, 24 carbapenemase-producing Kpn strains, most of them (23/24) carrying *bla*_OXA-48_, isolated from urine samples of nosocomial or community origin in northwest Spain, were characterized in terms of antibiotic resistance, virulence traits and biofilm-forming capacity to identify UTI-associated traits. Additionally, the susceptibility to bacteriophages, both in planktonic cultures and biofilms, was assessed

**Results and discusion:**

High levels of resistance to other antibiotics were also observed, with 83.3% of strains classified as multidrug-resistant. Most isolates belonged to ST15 (*n* = 11) and ST147 (*n* = 8), representing high-risk clones, all of them harboring *bla*_OXA-48_. Strain 6022, belonging to ST307, represented a third high-risk clone. Principal component analysis (PCA) revealed clustering of strains into two main groups according to antibiotic resistance and virulence genes, corresponding to the two predominant STs. None of the urine strains carried hypervirulence-associated genetic markers, despite the high proportion of hypermucoviscous isolates. Six lytic bacteriophages belonging to the genera *Webervirus*, *Jiaodavirus*, and *Druslisvirus*, were evaluated against the Kpn isolates. All six phages lysed at least one strain. None of the phages lysed ST15 isolates, confirming previously reported specificity associated with ST and capsule locus types. The combination of *Webervirus* kpv33d1 and *Jiaodavirus* kpv33d6 completely inhibited planktonic growth of selected strains, even at low multiplicities of infection (MOI = 0.01). Importantly, this low MOI was sufficient to significantly reduce colony-forming unit counts in preformed biofilms, a key factor in infection persistence and antibiotic resistance in Kpn. These results indicate that some genomic and phenotypic traits seem to be associated with UTI Kpn strains, even though a higher number of strains should be analyzed. The isolation of wide-spectrum phages for the treatment of Kpn UTI infections is demonstrated to be feasible, but our results emphasize the need to assess biofilm-disrupting activity when selecting phages for therapeutic applications against *K. pneumoniae*.

## 1 Introduction

Urinary Tract Infections (UTIs) constitute one of the most common bacterial infections, with an estimated 405 million infections and 267,000 deaths in 2019 worldwide (Yang et al., 2022), and are a major cause of female morbidity. The opportunistic pathogen *Klebsiella pneumoniae* (Kpn) is the second most frequent cause of uncomplicated UTI after uropathogenic *Escherichia coli* (UPEC), accounting for 6–10% of community-acquired and nosocomial UTIs (Foxman, 2010; Timm et al., 2025), but in terms of antibiotic resistance, Kpn strains represent the greatest concern in UTI (Sati et al., 2025).

The increasing prevalence of multidrug-resistant (MDR) Kpn strains, resulting from the accumulation of horizontally acquired genes, including extended-spectrum *β*-lactamases (ESBLs) and/or carbapenemases, as well as mutations in chromosomal genes (Lam et al., 2021), has turned previously simple infections such as UTIs into difficult-to-treat conditions. Moreover, the ability of Kpn to form biofilms further contributes to persistence and reduced treatment efficacy. The World Health Organization has accordingly included Kpn in the Priority 1 group of ESKAPE microorganisms for which there is an urgent need to develop new therapeutic strategies (World Health Organization, 2017; Tacconelli et al., 2018). Furthermore, the most recent WHO report places carbapenemase-producing Kpn (CP-Kpn) among the top priority microorganisms for antibiotic resistance surveillance and development of novel antimicrobial strategies (Sati et al., 2025).

In addition to the high prevalence of multidrug-resistant (MDR) isolates among nosocomial classical Kpn strains (cKpn), a new group of hypervirulent Kpn (hvKp) strains, capable of infecting healthy individuals and showing an increased tendency to be invasive, is increasingly reported in Kpn infections (Zhu et al., 2021). Moreover, the emergence of convergent strains that are MDR and present the high invasive traits of hypervirulent strains is of great concern (Gonzalez-Ferrer et al., 2021).

While the factors involved in the pathogenesis of *E. coli* strains causing urinary tract infections (UPEC) have been widely studied (Terlizzi et al., 2017; Zhou et al., 2023), and specific genes have been associated with an enhanced capability to colonize the urinary tract (Spurbeck et al., 2012), much less is known about the specific genomic and phenotypic characteristics of UTI-related Kpn strains and their relationship with AMR profiles. Virulence factors such as Type I and Type 3 pili, capsule production, and siderophore systems have been associated with UTI Kpn strains (Li et al., 2014; Flores-Mireles et al., 2015; Paczosa and Mecsas, 2016).

Some reports suggest differential features of UTI-related isolates. A comparison between Kpn isolates obtained from patients with bacteraemia and UTI found differences in capsular types and in the capsule-related gene *htrA*, which was more frequently detected in urine isolates than in blood isolates, although the number of bacteraemia isolates analyzed was low compared with urine isolates (Kao et al., 2023). Similarly, Kpn isolates from patients with community-acquired bloodstream infection exhibited higher resistance to antimicrobial agents than isolates from community-acquired UTI, pointing to differently adapted populations (Monteiro et al., 2025).

In contrast, several studies revealed that environmental and clinical Kpn isolates are indistinguishable based on phenotypic and genotypic characterization (Gholizadeh et al., 2024; Abbas et al., 2025). Moreover, although carbapenemases have been associated with certain multilocus sequence types, mainly ST258 and its related variants such as ST11 (Pitout et al., 2015; Tryfinopoulou et al., 2023), no specific lineage has been clearly linked to UTI among carbapenem-resistant strains. There is therefore a need to phenotypically and genetically analyze the traits that characterize UTI-associated Kpn strains, particularly MDR isolates, and to assess whether these characteristics differ between strains of nosocomial or community origin.

In parallel, alternative therapeutic approaches are being explored to address the increasing burden of MDR Kpn infections. Among these, bacteriophage therapy has emerged as a promising strategy for infections caused by antimicrobial-resistant strains (Hatfull et al., 2022; Kondo et al., 2023), and clinical studies suggest its safety and potential efficacy, particularly in combination with antibiotics (Eskenazi et al., 2022; Uyttebroek et al., 2022; Pirnay et al., 2024). Phage therapy presents advantages over conventional antibiotics, including high host specificity, biofilm penetration capacity, and limited impact on the microbiota (Hatfull et al., 2022; Uyttebroek et al., 2022). However, therapeutic phages must lack integrase genes, antibiotic resistance determinants, and toxin-encoding genes, and challenges remain regarding formulation and stabilization for clinical use (Vandenheuvel et al., 2015; Principi et al., 2019). Although specificity may require tailored phage selection, strategies such as phage cocktails and co-evolutionary adaptation can broaden host range and may promote antibiotic re-sensitization in resistant isolates (Georjon and Bernheim, 2023; Chen et al., 2024; Pirnay et al., 2024).

In the context of UTIs, phage therapy may be particularly attractive due to the possibility of local intravesical administration and the potential to tackle biofilm-associated infections. Although clinical experience in UTIs remains limited and randomized controlled trials are scarce (Leszczyński et al., 2006; Ujmajuridze et al., 2018; Kuipers et al., 2019; Bao et al., 2020; Le et al., 2023), intravesical bacteriophage therapy with a commercial phage cocktail was shown to be non-inferior to standard antibiotic treatment, although not superior to placebo bladder irrigation (Leitner et al., 2021), highlighting the importance of selecting phages with an adequate lytic spectrum.

Recently, numerous studies have focused on the isolation of bacteriophages specific to Kpn strains (Gholizadeh et al., 2024; Abbas et al., 2025). However, the presence of capsule, which often serves as a phage receptor, may restrict host range (Dunstan et al., 2023; Lourenço et al., 2023; Pacios et al., 2023). Moreover, given the strong biofilm-forming capacity of many Kpn strains, the identification of phages capable of disrupting preformed biofilms is of particular relevance, which would also facilitate antibiotic penetration (Mulani et al., 2022; Das and Kaledhonkar, 2024; Karthika et al., 2025; Samanta and Datta, 2025). Therefore, in this study, we aimed to describe the resistance profile, virulence gene content, and biofilm-forming capacity of a collection of 24 carbapenemase-producing *K. pneumoniae* strains, most of them OXA-48, isolated from urinary tract infections of nosocomial and community origin, in order to try to identify genomic and phenotypic traits that could be associated with urinary tract infection capacity. Moreover, their susceptibility to bacteriophage infection (both in planktonic growth and in preformed biofilms) was also assessed.

## 2 Materials and methods

### 2.1 Bacterial isolates and antimicrobial susceptibility testing

This study included 24 carbapenemase-producing *K. pneumoniae* (CP-Kpn) strains, most of them producing OXA-48, isolated from urine samples between 2014 and 2022 at the Microbiology Laboratory of the Vigo University Hospital Complex (CHUVI, Spain). All strains had been checked for carbapenemase gene presence by PCR and antibiotic resistance profile upon isolation. Strains were selected in order to obtain a representative sample of the most diverse antibiotic resistance profile among the CP-Kpn strains available for the period. The origin of infection (community or nosocomial) and type of sample collection (midstream, indwelling catheter or nephrostomy) were recorded. Isolates from nursing home residents were classified as nosocomial due to the high frequency of hospital admission among these patients. Strains were stored at −80 °C in cryotubes (CRYOINSTANT-deltalab REF:409113/6). The identification of the selected strains was confirmed by MALDI-TOF MS (Bruker Daltonik, Bremen, Germany), carbapenemase production was confirmed using the O. K. N. V. I. RESIST-5 immunochromatographic assay (Coris Bioconcept) and Minimum Inhibitory Concentrations (MICs) were further confirmed by broth microdilution using the MicroScan Walkaway (Beckman Coulter) with the NEG MIC 57 panel (REF c58012), which provided MIC for a higher number of antibiotics than originally tested. Results were interpreted according to EUCAST v13.0 clinical breakpoints for Enterobacterales. Isolates were classified as multidrug-resistant (MDR) when resistant to at least one agent in three or more antimicrobial categories (Magiorakos et al., 2012).

### 2.2 Genomic analysis

Whole genome sequencing (WGS) was performed to identify antimicrobial resistance and virulence genes, determine multilocus sequence types (MLST), capsular types, and detect phage-resistance mechanisms. Genomic DNA was extracted using the QIAsymphony DSP Virus/Pathogen Midi kit (Qiagen, Hilden, Germany). Libraries were constructed using Illumina DNA Prep (Illumina, San Diego, United States) and sequenced on an Illumina MiSeq platform (MiSeq Reagent kit v3, 2 × 300 cycles). Reads were assembled *de novo* using Pathogenwatch.^1^ This Whole Genome Shotgun project has been deposited at DDBJ/ENA/GenBank under the accession JBWJIH000000000-JBWJJE000000000 (BioProject PRJNA1438902). The version described in this paper is version JBWJIH010000000-JBWJJE010000000.

Sequence types (STs) were assigned using the *K. pneumoniae* BIGSdb database (Institut Pasteur; Diancourt et al., 2005).^2^ The cgMLST (core genome MLST) of all isolates was performed using chewBACCA (Silva et al., 2018). The cgMLST scheme was obtained from the *K. pneumoniae sensu lato* strain containing 2,538 loci,^3^ and the NTUH-K2044 strain was included as a reference. The chewBACCA software was used for distance matrix and phylogenetic tree generation. The phylogenetic tree was plotted and visualized with Microreact.^4^

Capsular (K) types were determined using Kaptive (Wick et al., 2018). Antimicrobial resistance genes were identified using Resfinder v.4.1^5^ (Zankari et al., 2012), the Comprehensive Antibiotic Resistance Database (CARD; McArthur et al., 2013),^6^ and Pathogenwatch (Kleborate-derived; Lam et al., 2021).^7^ Plasmid replicons were identified with PlasmidFinder (Carattoli et al., 2014). Twenty-three virulence-associated genes (Supplementary Table 1) were screened using BLAST (>95% identity threshold) with sequences retrieved from BIGSdb (Jolley et al., 2018) and NCBI GenBank. Phage-resistance mechanisms were identified by DefenseFinder, a bioinformatic program designed for the systematic identification of known antiphage defense mechanisms (Tesson et al., 2022).

### 2.3 Phenotypic characterization

#### 2.3.1 Hypermucoviscosity

The hypermucoviscous (HMV) phenotype was evaluated using a modified string test (Shon et al., 2013). Because culture conditions influence the result of the string test in *K. pneumoniae*, isolates were evaluated on LB medium and LB supplemented with 0.4% glucose (LB + G) and incubated at 37 °C for 24 h under microaerophilic conditions (Silva-Bea et al., 2024b). A strain was considered HMV-positive when a viscous string ≥5 mm was formed after lifting a colony with a loop.

#### 2.3.2 Biofilm formation and quantification

Biofilm culture was assessed using the active attachment (AA) method, which promotes adhesion-dependent biofilm development while allowing media changes without disturbing the biofilm structure (Exterkate et al., 2010; Silva-Bea et al., 2024b). Biofilms were grown in triplicate in LB broth supplemented with 0.4% glucose on 18 × 18 mm glass coverslips. Coverslips were fixed to a custom-designed aluminium lid fitted to a standard 12-well plate and partially immersed in the culture medium. Wells were inoculated with bacterial suspensions adjusted to an optical density at 600 nm (OD₆₀₀) of 0.05, measured with a Spectronic Genesys 5 spectrophotometer, and incubated at 37 °C for 24 h under microaerophilic conditions (CampyGEN 2.5 L, REF: CN0025A). Biofilm biomass was quantified by 0.04% crystal violet staining. After washing and drying, the bound dye was solubilized with 33% acetic acid and measured at 590 nm. The OD₆₀₀ of the cultures was also measured to normalize biofilm biomass to total bacterial growth (Peeters et al., 2008). Strains were classified using tertiles as follows: low former (Abs_590nm_ < 0.47), moderate former (0.47 < Abs_590nm_ < 0.89), and high biofilm former (Abs_590nm_ > 0.89; Cusumano et al., 2019).

### 2.4 Isolation and genomic characterization of bacteriophages

Phages were isolated from urban and hospital wastewater samples using a double-layer agar method following enrichment with two *K. pneumoniae* strains (ATCC 13883^T^ and KLEB-33) as hosts. KLEB-33 is an MDR, carbapenem-resistant, hyper-biofilm-forming, HMV, clinical isolate belonging to ST16, harboring hypervirulence genes (Silva-Bea et al., 2024a). Both strains produce capsule, which is thicker in the type strain. For phage enrichment, wastewater (50 mL) was mixed with 50 mL double-strength Trypticase soy Broth (TSB) medium and inoculated with 5 mL of an overnight culture of each host strain, followed by incubation at 37 °C for 48 h. After centrifugation at 6800xg for 15 min, supernatants were filtered through 0.22 μm filters. Phages were isolated by mixing serial dilutions of the filtrate with host bacteria (OD₆₀₀ 0.5–0.6), embedding in soft Luria-Bertani (LB) agar (0.8%), and incubating at 37 °C for 24 h. Plaques were picked, propagated, and purified by repeated plating. Phage titers (PFU/mL) were determined by the double agar method. Phage stocks were stored at −80 °C in 30% glycerol or at 4 °C, and titers were verified before use. Of the 22 isolated phages, six were selected to evaluate lytic activity against the 24 UTI isolates. Five were obtained using KLEB-33 and one using ATCC 13883^T^ as enrichment hosts.

Viral DNA was extracted using the QIAsymphony kit and sequenced on an Illumina iSeq platform. Assemblies were generated *de novo,* and viral genera were assigned using Pathogenwatch. The 20 closest reference genomes (NCBI BLAST) were retrieved for phylogenetic analysis. Alignments were performed with MAFFT and phylogenetic trees were constructed in MEGA using the Neighbor-Joining method with the Jukes-Cantor model. Phage family assignment was performed using PhaGCN (Shang et al., 2021). Resfinder 4.1 and Virulence Finder 2.0 were used to screen for resistance and virulence genes. Phage lifecycle was predicted using PhaTYP (Shang et al., 2023). Genome length and GC content were obtained from Pathogenwatch. DefenseFinder was used to identify phage genetic defense mechanisms. Phage polysaccharide depolymerase genes were searched with BLASTx using all depolymerase entries in UniProt Knowledgebase (UniProtKB) reviewed database. Results with E-value <0.0001 and identity >80% were considered positive. All FASTA files generated were deposited in GenBank (accession numbers PZ324420-PZ324425).

### 2.5 Phage susceptibility assays

Initial screening of phage susceptibility was performed with the six selected phages and the 24 Kpn strains using the spot test method (Kutter, 2009). Soft LB agar inoculated with bacterial cultures (OD₆₀₀ 0.5–0.6) was poured onto square LB plates (120 × 120 mm). Serial phage dilutions (10^−1^ to 10^−10^) prepared in phosphate buffer (pH 7) were spotted (5 μL) and plates incubated at 37 °C for 24 h. Lysis zones were then recorded. Productive infection was confirmed using the standard double agar plaque assay to distinguish true lytic activity from non-productive lysis (e.g., lysis from without; Abedon, 2011; Daubie et al., 2022). Efficiency of plating (EOP) was calculated as the ratio of average PFU on the test strain to average PFU of the reference host (Kutter, 2009; Khan Mirzaei and Nilsson, 2015).

Liquid infection assays were performed in microtiter plates using strain 2173 and phages *Webervirus* kpv33d1 and *Jiaodavirus* kpv33d6 as previously described (Xie et al., 2018). Strain 2173 was selected as the most phage-sensitive isolate. *Jiaodavirus* kpv33d6 was chosen due to its highest activity against strain 2173, whereas *Webervirus* kpv33d1 showed the broadest host range. Cultures were adjusted to OD₆₀₀ 0.01 (~10^6^ CFU/mL) and infected at multiplicities of infection (MOI) of 0.1, 1, and 10 in 96-well U-bottom plates. Plates were sealed with a gas-permeable membrane (Breathe-Easy®, Z380059) and incubated at 37 °C for 24 h, with OD₆₀₀ measured every 30 min.

Biofilm susceptibility of strains KLEB-33 and 2173 to *Webervirus* kpv33d1 and *Jiaodavirus* kpv33d6 was evaluated using 24 h pre-formed biofilms (AA method). After washing with sterile phosphate buffer to remove non-adherent cells, coverslips were transferred to fresh 12-well plates containing phosphate buffer supplemented with phage at 10^6^ PFU/mL (MOI 0.01). Plates were incubated at 37 °C for an additional 24 h. Biofilms were detached by vortexing coverslips for 15 s in phosphate buffer and viable bacteria quantified by CFU enumeration.

### 2.6 Statistical analysis

Principal component analysis were performed using RStudio version 2021.09.0. Differences in biofilm formation according to strain origin, ST, or HMV phenotype, as well as phage efficacy in preformed biofilms, were assessed using a Student’s t-test (*p* < 0.001).

## 3 Results

### 3.1 Bacterial isolates and antibiotic susceptibility

Most of the 24 carbapenemase-producing *K. pneumoniae* isolates recovered from urinary tract infections (23/24) were OXA-48 producing strains, according to the immunochromatographic assay. 17 were of nosocomial origin, including hospital and nursing home facilities, and the remaining seven were community-acquired. Most isolates were obtained from midstream urine samples (18/24), five from indwelling catheters, and one from a nephrostomy (Supplementary Table 2).

**Figure 1:**
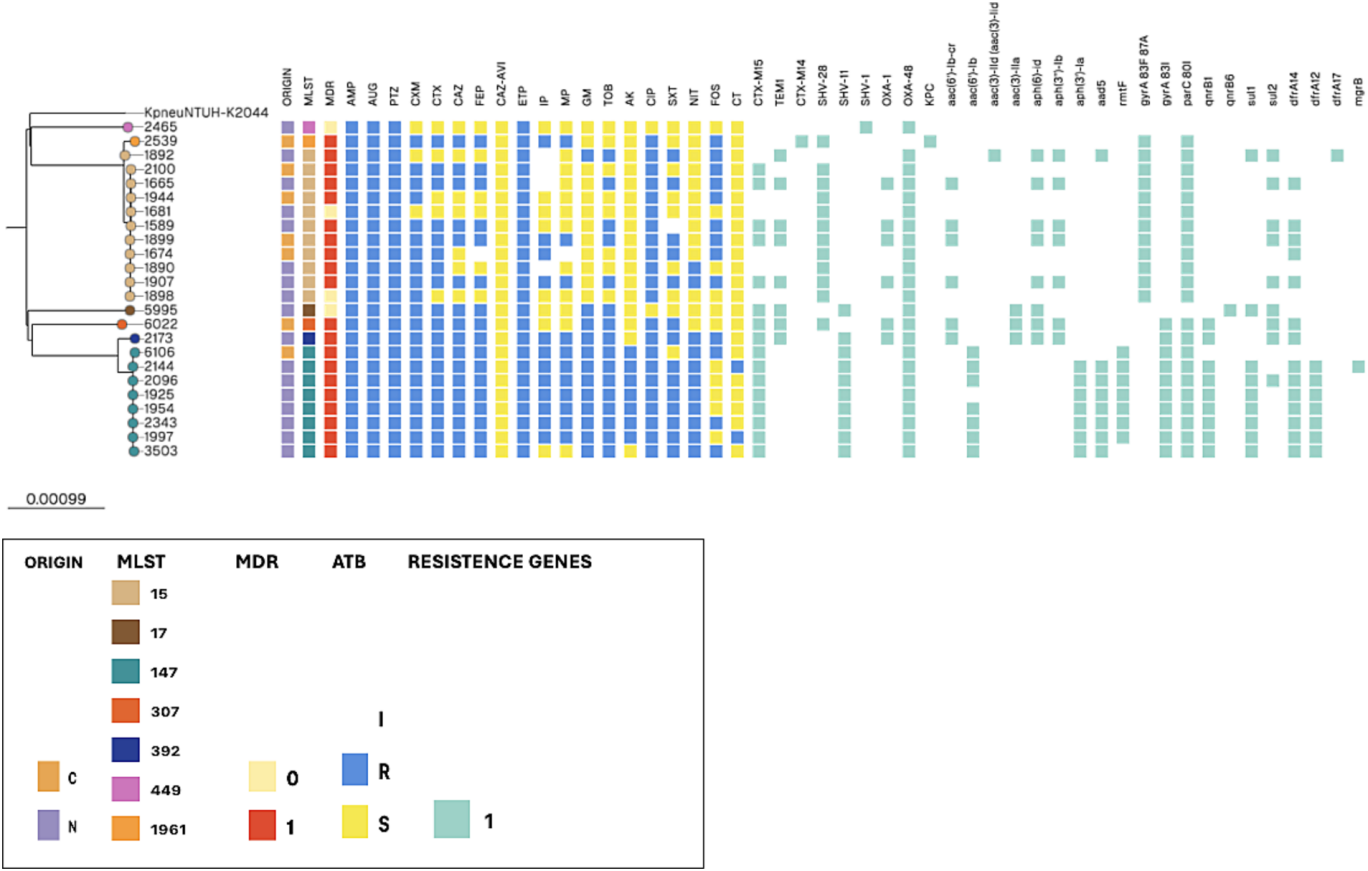
Phylogenetic tree of the 24 carbapenemase-producing *K. pneumoniae* strains isolated from urinary tract infection samples used in this study based on core genomic multilocus strain type analysis (cgMLST) and multidrug-resistance (MDR: red), susceptibility to antibiotics (R, resistant: blue, S, sensitive: yellow, I, intermediate: white) and presence of antibiotic resistance genes (presence: green). Information on the origin of the strains (N: nosocomial: purple color, C: community: orange color) is also presented.

**Figure 2:**
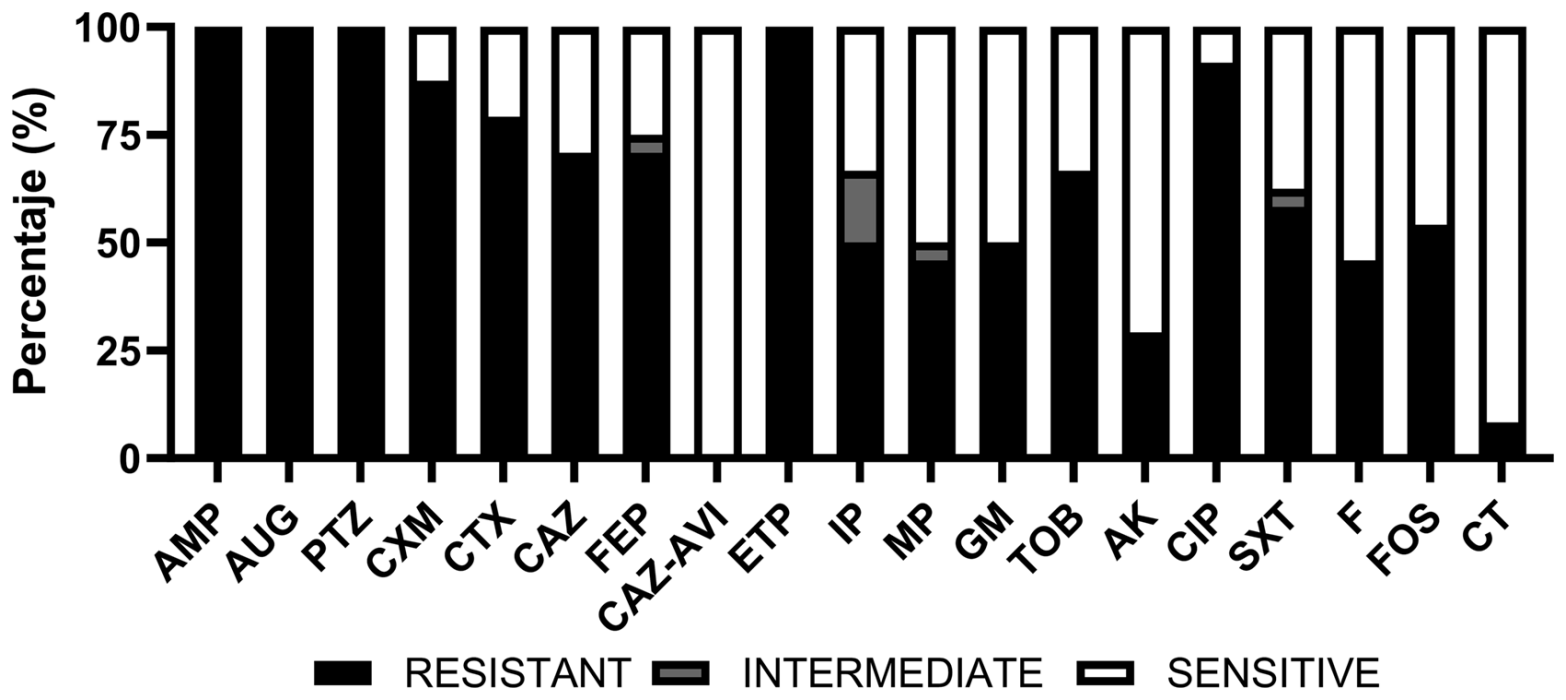
Susceptibility to antibiotics (%), according to EUCAST v13.0 clinical breakpoints for *Enterobacterales* in the 24 carbapenemase-producing *K. pneumoniae* strains isolated from urinary tract infection samples.

Individual phenotypic resistance profiles against the 15 tested antibiotics are shown in Figure 1. All isolates were resistant to ampicillin, amoxicillin, clavulanic acid, and piperacillin-tazobactam. 87.5% were resistant to cefuroxime, 79.2% to cefotaxime, 70.8% to ceftazidime, and 70.8% to cefepime. However, all strains were sensitive to ceftazidime avibactam. *In vitro* resistance to carbapenems was 50% for imipenem, 45.8% for meropenem, and 100% for ertapenem. With respect to aminoglycosides, 50% of strains were resistant to gentamicin, 66.6% to tobramycin, and 29.2% to amikacin. 91.6% were resistant to ciprofloxacin, 58.3% to trimethoprim sulfamethoxazole, 45.8% to nitrofurantoin, and 54.2% to fosfomycin. Resistance to colistin was observed in only two strains. 83.3% of the strains were MDR (Figure 2).

### 3.2 Genomic characterization

MLST analysis identified 7 different sequence types (STs), the most frequent being ST15 (11 strains), followed by ST147 (8 strains) (Figure 1). The remaining STs were observed in a single isolate: ST392, ST1961, ST449, ST17, and ST307. All ST147 and ST15 strains belonged to high-risk clones because they all carried the *bla*_OXA-48_ gene. Moreover, strain 6022 represented a third high-risk clone (ST307/OXA-48; Pitout et al., 2019; Cañada-García et al., 2022; Figure 1). A strong correlation between KL type and ST was observed: 10 out of the 11 ST15 isolates were KL112, and all ST147 isolates were KL64 (Table 1). The capsule types KL24, KL27, KL48, KL22, KL25, and KL102 were present in a single strain.

**Table 1:** MLST (multilocus sequence types), genomic capsular determinant (K locus), hipermucoviscosity phenotype (HMV) (0: no HMV phenotype, 1: HMV in LB only, 2: HMV in LB + G only, 3: HMV in both LB and LB + G), biofilm (L: low former, M: moderate former, H: high former) and presence (X) of main virulence genes in the 24 carbapenem-resistant Kpn strains isolated for urinary tract infection samples used in this study.

ID strain			1589	1681	1665	1674	1890	1892	1898	1899	1907	1944	2100	5995	1954	1925	1997	2096	2144	2343	6106	3503	6022	2173	2465	2539
MLST			15	15	15	15	15	15	15	15	15	15	15	17	147	147	147	147	147	147	147	147	307	392	449	1961
K locus			*112*	*112*	*112*	*112*	*112*	*24*	*112*	*112*	*112*	*112*	*112*	*25*	*64*	*64*	*64*	*64*	*64*	*64*	*64*	*64*	*102*	*27*	*22*	*48*
HMV			*3*	*0*	*1*	*0*	*0*	*2*	*2*	*0*	*2*	*2*	*0*	*0*	*3*	*3*	*3*	*2*	*2*	*2*	*0*	*2*	*0*	*2*	*3*	*0*
BIOFILM			*L*	*H*	*M*	*L*	*H*	*L*	*H*	*M*	*H*	*H*	*M*	*M*	*L*	*L*	*M*	*L*	*H*	*M*	*H*	*L*	*M*	*M*	*H*	*H*
Virulence factors	Phenotype	Gene																								
	Mucoviscosity	*rmpA2*																								
	peg 344	*peg-344*																								
	Colibactin	*clb ABCDEFGHIJKLMNOPQR*																								
	Salmochelin	*iroBCDN*																								
	Aerobactin	*iucABCD*																								
		*iutA*	X	X	X	X	X	X	X	X	X	X	X	X	X	X	X	X	X	X	X	X	X	X	X	X
	Enterobactin	*entABCDEF*	X	X	X	X	X	X	X	X	X	X	X	X	X	X	X	X	X	X	X	X	X	X	X	X
		*fepABCDG*	X	X	X	X	X	X	X	X	X	X	X	X	X	X	X	X	X	X	X	X	X	X	X	X
	Yersiniabactin	*ybt*A	X	X	X	X	X	X	X	X	X	X	X	X	X	X	X	X	X	X			X		X	
		*ybtE*	X	X	X	X	X	X	X	X	X	X	X	X	X	X	X	X	X	X		X	X			
		*ybtP*	X	X	X	X	X	X	X	X	X	X	X	X	X	X	X	X	X	X		X	X			
		*ybtQ*	X	X	X	X	X	X	X	X	X	X	X	X	X	X	X	X	X	X		X	X		X	
		*ybtS*	X	X	X	X	X	X	X	X	X	X	X	X	X	X	X	X	X	X		X	X		X	
		*ybtT*	X	X	X	X	X	X	X	X	X	X	X	X	X	X	X	X	X	X		X	X		X	
		*ybtU*	X	X	X	X	X	X	X	X	X	X	X	X	X	X	X	X	X	X		X	X		X	
		*ybtX*	X	X	X	X	X	X	X	X	X	X	X	X	X	X	X	X	X	X		X	X		X	
		*irp1*	X		X			X	X	X	X	X	X	X	X	X	X	X	X	X		X	X		X	
		*irp2*	X	X	X		X	X	X	X	X	X	X	X	X	X	X	X		X		X	X		X	
		*fyu*A	X	X	X	X	X	X	X	X	X	X	X	X	X	X	X	X	X	X		X	X		X	
	Iron uptake	*kfu*A	X	X	X	X	X	X	X	X	X	X	X												X	X
		*kfu*B	X	X	X	X	X	X	X	X	X	X	X												X	X
		*kfu*C	X	X	X	X	X	X	X	X	X	X	X							X				X	X	X
	Tellurite resistance	*terABCDEWXYZ*																								X

	Capsule synthesis	*rcs*A	X	X	X	X	X	X	X	X	X	X	X	X	X	X	X	X	X	X	X	X	X	X	X	X
		*rcs*B	X	X	X	X	X	X	X	X	X	X	X		X	X	X	X	X	X	X		X	X		
		*rcs*C	X	X	X	X	X	X	X	X	X	X	X	X	X	X	X	X	X	X	X	X	X	X	X	X
		*rcs*D	X	X	X	X	X	X	X	X	X	X	X	X	X	X	X	X	X	X	X	X	X	X	X	X
		*rcs*F	X	X	X	X	X	X	X	X	X	X	X	X	X	X	X	X	X	X	X	X	X	X	X	X
		*kvg*AS																							X	
	Type 3 Fimbriae	*mrk*A	X	X	X	X	X	X	X	X	X	X	X	X	X	X	X	X	X	X		X	X	X	X	X
		*mrk*B	X	X	X	X	X	X	X	X	X	X	X	X	X	X	X	X	X	X		X	X	X	X	X
		*mrk*C	X	X	X	X	X	X	X	X	X	X	X	X	X	X	X	X	X	X		X	X	X	X	X
		*mrk*D	X	X	X	X	X	X	X	X	X	X	X	X	X	X	X	X	X	X	X	X	X	X	X	X
		*mrk*F	X	X	X	X	X	X	X	X	X	X	X	X	X	X	X	X	X	X	X	X	X	X	X	X
		*mrk*H	X	X	X	X	X	X	X	X	X	X	X	X	X	X	X	X	X	X	X	X	X	X	X	X
		*mrk*I	X	X	X	X	X	X	X	X	X	X	X	X	X	X	X	X	X	X	X	X	X	X	X	X
		*mrk*J	X	X	X	X	X	X	X	X	X	X	X	X	X	X	X	X	X	X	X	X	X	X	X	X
	Type 1 Fimbriae	*fimABCDEFGHI*	X	X	X	X	X	X	X	X	X	X	X	X	X	X	X	X	X	X		X	X	X	X	X
	Fimbrial adhesin	*kpiABCDEFG*													X	X	X	X	X	X	X	X	X	X		
	Microcin	*mce*A																							X	
		*mce*B																							X	
		*mce*C																							X	
		*mce*D																							X	
		*mce*E																							X	
		*mce*G																							X	
		*mce*H																							X	
		*mce*I																							X	
		*mce*J																							X	
	Allantoin Metabolism	*allABCDRS*																								
		*ybb*W																								
Stress response	*htr*A	X	X	X	X	X	X	X	X	X	X	X	X	X	X	X	X	X	X	X	X	X	X	X	X

As expected, all positive strains in the immunochromatographic assay harbored the carbapenemase *bla*_OXA-48_ gene. Strain 2539 (ST1961), which was the only OXA-48 negative strain, carried *bla*_KPC-2_ (Figure 1). The most frequent extended-spectrum *β*-lactamase (ESBL) gene was *bla*_CTX-M-15_ (16 strains), followed by *bla*_SHV-28_ (12), *bla*_SHV-11_ (10) and *bla*_CTX-M-14_ (1). The *bla*_SHV-1_ gene was detected in only one strain. Six isolates also carried the *bla*_OXA-1_ narrow-spectrum oxacillinase.

Aminoglycoside resistance genes were detected in 16 strains, including N-acetyltransferases (*aac(6′)-Ib-cr, aac(6′)-Ib, aac(3)-IId, aac(3)-IIa*) and/or O-phosphotransferases (*aph(6)-Id, aph(3″)-Ib, aph(3″)-Ia). aac(6′)-Ib-cr, aph(6)-Id and aph(3″)-Ib* were detected in ST15 strains, whereas *acc(6′)-Ib, aph(3′)-Ia* and the 16S rRNA methyltransferase *rmtF* were found in ST147 strains.

**Figure 3:**
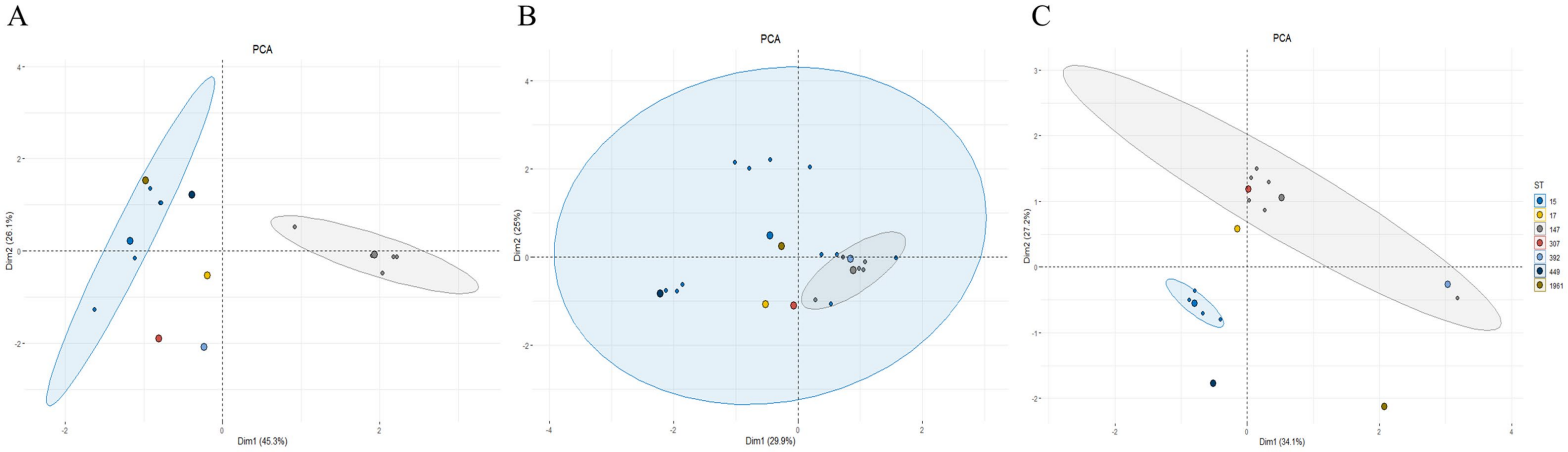
Principal component analysis of the antibiotic resistance genes (ARGs) profile **(A)**, phenotypic resistance profile **(B)**, and virulence genes **(C)** of the 24 carbapenemase-producing *K. pneumoniae* strains isolated from urinary tract infection samples.

Plasmid-mediated *qnr*-like quinolone resistance determinants were detected in 10 isolates: nine carried *qnrB1* (mostly ST147) and one *qnrB6* (ST17). Fluoroquinolone resistance mutations in *gyrA* (83F and 87A) were present in 12 isolates (all ST15), and 83I in 10 strains (8 ST147). Mutations in *parC* (80I) were detected in 22 strains. Genes conferring trimethoprim-sulfamethoxazole resistance were identified in 16 strains, with several strains carrying more than one gene: *sul1* (9), *sul2* (9), *dfrA14* (14), *dfrA12* (7, all ST147), and *dfrA17* (1 strain). Truncation or loss of *mgrB* was detected in one of the two phenotypically colistin-resistant isolates (strain 2144). The complete resistome is shown in Figure 1. Interestingly, while principal component analysis (PCA) separated strains into two main groups according to antibiotic resistance gene (ARG) profiles, corresponding to ST15 and ST147 (Figure 3A), no clear differentiation was observed when phenotypic resistance profiles were analyzed (Figure 3B). Moreover, no association with origin or sampling method was detected.

The most prevalent plasmid replicon was IncL (20 strains), all carrying the *bla*_OXA-48_ gene. IncR was detected in 17 strains, 13 harboring *bla*_CTX-M-15_. Plasmids repB(R1701; 15 strains) and IncFIB(K; 11 strains) were also common. ST15 and ST147 strains shared IncL, IncR, and repB(R1701), whereas IncFIB(K) and CoIRNAI were specific to ST15, and IncFII(pKPX1) to ST147. Strain 2539 (ST1961) showed a distinct plasmid profile (Supplementary Table 3).

Twenty-three phage defense systems were identified. Adaptive systems included CRISPR-Cas, innate mechanisms included restriction-modification (RM) systems, and abortive infection systems such as Abi, Lamassu, SEFIR. and PrrC mechanisms. Additional systems included Hachiman, Gabija, BREX CBASS Retron, Gao, Pd-T, Shango, Rst_TIR-NLR, Septu, Pif, Druantia, Rosmer TA, and Zorya. Several toxin-antitoxin (TA) systems were found, including: Hok/Sok, MazEF, and Sana. Abi, CRISPR-Cas, MazEF and RM systems were widely distributed. ST15 shared a common defense profile, whereas ST147 strains uniquely carried PD-T and Pif systems (Supplementary Table 4).

All isolates harbored genes involved in the synthesis of the siderophore enterobactin (*entABCDEF*) and its corresponding receptor (*fepABCDG*). Regarding yersiniabactin biosynthesis, the complete *ybtAEPQSTUX* cluster was detected in 79.1% of the strains, three isolates lacked the complete cluster, and two lacked parts of the genes. The complete irp12 cluster was present in 70.8% of the strains, and 87.5% of the strains carried its outer membrane receptor (*fyuA*). The genes involved in salmochelin (*iroBCDN)* and aerobactin (*iucABCD*) biosynthesis were absent in all strains; however, the aerobactin receptor gene (*iutA*) was detected in all isolates. Another iron acquisition-associated virulence factor cluster, *kfuABC*, was fully present in 54.1%, the cluster was completely absent in nine isolates and was partially present in two isolates. Interestingly, most strains within ST147 lack the *kfuABC* cluster (7 out of 8).

Regarding capsule-associated virulence factors, the *rcsABCDF* cluster was complete in 83.3% of strains, while *kvgAS* was detected in a single isolate (ST449). None of the 24 strains carried a mucoid phenotype regulator gene (*rmpA, rmpA2*), the metabolic transporter *peg-344*, the allantoin metabolism clusters (*allABCDRS* and *ybbW*), or the colibactin cluster (*clbABCDEFGHIJKLMNOPQR*).

Virulence genes involved in the synthesis of type 1 (*fimABCDFGHI*) and type 3 (*mrkABCDEHIJ*) fimbriae were found in 95.8 and 100% of strains, respectively. In the case of type 3 fimbriae, only one strain did not possess the complete operon. The chaperone-usher pili system *kpiABCDEFG*, previously associated with the globally disseminated high-risk clone ST15, was carried by the 8 isolates belonging to ST 147 and the two representatives from ST392 and ST307.

The heavy metal resistance operon *terABCDEWYZ*, associated with hypervirulent strains (Passet and Brisse, 2015), and the microcin E492–associated operon *mceABCDEGHIJ* were each detected in a single strain (ST1961 and ST449, respectively). Finally, the virulence-related periplasmic serine endoprotease DegP-like gene (*htrA*) was present in all isolates (Table 1).

Similar to ARG profiles, the PCA of the virulence genes separated strains into two main groups according to the predominant STs, namely ST15 and ST147 (Figure 3C). No effect of origin or mode of sample collection was observed (data not shown).

### 3.3 Phenotypic characterization

#### 3.3.1 Hypermucoviscous phenotype

**Figure 4:**
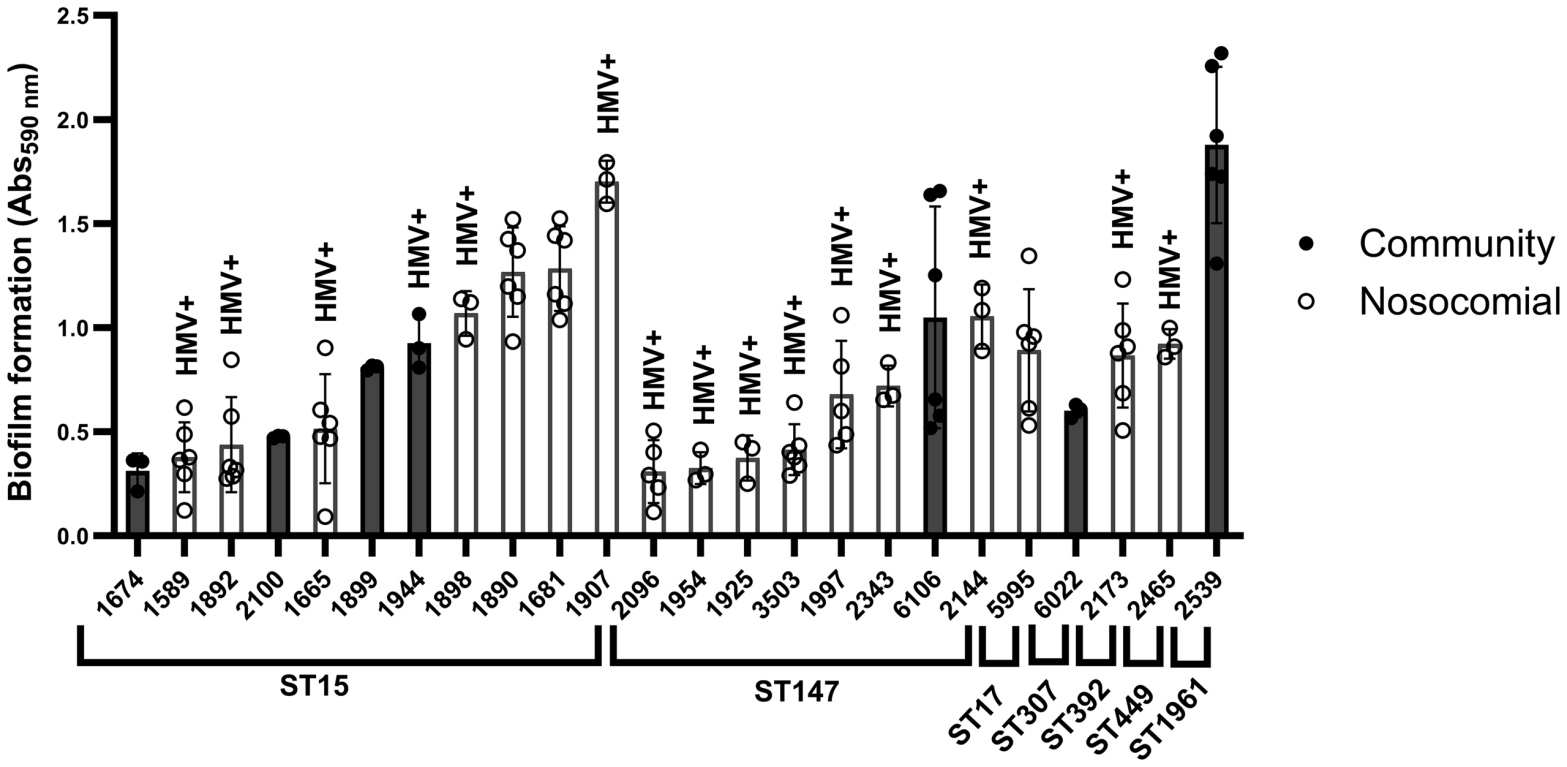
Biofilm-forming capacity in the active attachment biofilm cultivation system, measured with the crystal violet stain, of the 24 carbapenem-resistant *Kpn* strains isolated from urinary tract infection samples (*n* = 3; for strains presenting high variability, the experiment was performed twice). Biofilms were cultivated in LB supplemented with glucose (0.4%) in microaerobic conditions. The hypermucoviscous phenotype is marked. Strains of nosocomial origin are marked in white, strains of community origin are marked in gray.

Of the 24 strains, 15 were HMV + (62.5%) in at least one of the conditions tested (Table 1; Figure 4). Five strains were positive in both media, nine only in LB + G, and only and one was HMV+ only in LB. 87.5% of the strains within ST 147 were HMV+, whereas only 54.5% within ST15 showed this phenotype. With respect to the origin of the samples, 14 of the 17 nosocomially acquired strains were HMV+, whereas only 1 of the 7 community isolates presented the HMV phenotype (Figure 4). According to PCA, the HMV phenotype was not associated with ST, ARGs, or virulence genes (data not shown).

#### 3.3.2 Biofilm forming capacity

Eight strains were moderate biofilm formers and nine were strong formers, as assessed in the AA biofilm cultivation method (Table 1; Figure 4). The biofilm-forming capacity was not correlated with the HMV phenotype or the origin of the strains (Student’s t-Test, *p* > 0.05). In contrast, slightly higher biofilm biomass values were observed for ST15 strains compared with strains belonging to ST 147 (Student’s t-Test, *p* = 0.04; Figure 4).

### 3.4 Phage sensitivity

A collection of 22 phage isolates was obtained from urban and hospital wastewater using the laboratory strain ATCC 13883^T^ (low-biofilm former, non-HMV, ST14) and the clinical strain KLEB-33 (MDR, hyperbiofilm former, HMV, ST16; Silva-Bea et al., 2024a) as hosts. All 11 phages isolated using KLEB-33 enrichment cultures were recovered from urban wastewater. In contrast, among the phages isolated using ATCC 13883^T^, four were recovered from urban wastewater and seven from hospital wastewater. Of the 11 phages isolated from ATCC 13883^T^ enrichment cultures, 10 also showed lytic activity against KLEB-33. Conversely, only two of the 11 phages isolated using KLEB-33 were able to lyse ATCC 13883^T^. Six bacteriophages were selected for further study, either because they were able to lyse both model Kpn strains used (*Jiaodavirus* kpv33d4, *Webervirus* kpv33d6, and *Jiaodavirus* kpvth1) or because they showed high lytic activity against the clinical strain (*Druslisvirus* kpv33s1, *Webervirus* kpv33d1, and *Webervirus* kpv33d7).

Phage genome sequencing revealed that the six selected phages belonged to three different families: *Drexlerviridae* (2), *Straboviridae* (3), and *Autographiviridae* (1), and three genera (*Webervirus*, *Jiaodavirus,* and *Drulisvirus*; Table 2). All displayed a strictly lytic life cycle and did not carry resistance genes or bacterial virulence genes. The phages were named following the recommendations of the International Committee on Taxonomy of Viruses (ICTV) as *Webervirus* kpv33d1, *Jiaodavirus* kpv33d4, *Jiaodavirus* kpv33d6, *Webervirus* kpv33d7, *Druslisvirus* kpv33s1, and *Jiaodavirus* kpvth1 (Turner et al., 2023). *Jiaodavirus* kpv33d6 and *Jiaodavirus* kpvTh1 were almost identical (99% identity), despite being isolated using different host strains and from different but connected sources: Th1 was isolated from a hospital wastewater effluent that joined the general wastewater effluent from Santiago de Compostela, Spain, before entering the general wastewater treatment plant. The genomic characteristics of the six lytic phages (family, genus, genome size, CG content, lifestyle, virulence genes, resistance genes, and genetic defense mechanism) are summarized in Table 2, and their taxonomic positions are shown in Supplementary Figure 1.

**Table 2:** Taxonomic position, genome characteristics, presence of genetic defense mechanisms, number of UTI CP-Kpn strains lysed, efficiency of plating (EOP, ratio of average PFU on the test strain to average PFU of the reference host) and plaque morphology of the 6 bacteriophages selected for this study.

		*Webervirus kpv33d1*	*Jiaodavirus kpv33d4*	*Jiaodavirus kpv33d6*	*Webervirus kpv33d7*	*Drulisvirus kpv33s1*	*Jiaodavirus kpvth1*
Initial host strain		KLEB-33	KLEB-33	KLEB-33	KLEB-33	KLEB-33	ATCC13883^T^
Family		*Drexiervirie*	*Straboviridae*	*Straboviridae*	*Drexierviride*	*Autographiviridae*	*Straboviridae*
Genus		*Webervirus*	*Jiaodavirus*	*Jiaodavirus*	*Webervirus*	*Drulisvirus*	*Jiaodavirus*
Genome size (bp)		48.748	166.101	166.086	48.750	44.933	166.086
GC content		51%	39.6%	39.6%	51%	54.3%	39.0.6%
Genetic defense mechanisms		−	Anti-CBASS Anti-RM Anti-TA	Anti-CBASS Anti-TA	−	−	Anti-CBASS Anti-TA
Number of UTI strains lysed		3	3	1	2	1	3
EOP	Kpn strain						
	2096	1.17E-11					3.92E-13
	6106		7.60E-09				
	2173	1.6E+00	7.6E+00	3.6E+01	2.7E-01		3.7E-01
	5995	3.9E-10					1.7E-14
6022		1.3E-09		1.8E-11	3.2E-09	
Titer against KLEB-33 (PFU/mL)		3.0E+12	1.8E+15	5.0E+12	1.5E+14	1.1E16	1.2E+16
Depolymerase genes		+	−	−	+	+	−
Morphology of lysis plaques		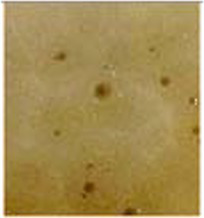	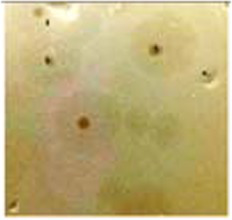	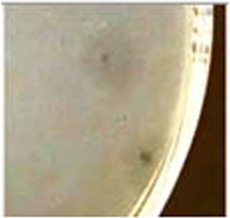	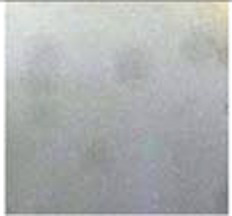	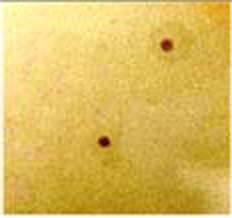	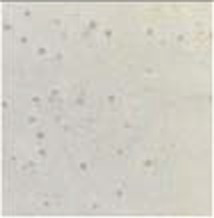

No lysogenic, bacterial virulence or antibiotic resistance genes were detected in their genomes. The accession numbers of the depolymerases found in the genomes of the phages are provided in the Supplementary Table 6.

Anti-CBASS (Cyclic oligonucleotide-Based Anti-phage Signaling System) and anti-TA (Toxin-Antitoxin) systems were detected in three phages, whereas anti-RM (Restriction and Modification) systems were detected in one. No anti-defense mechanisms were identified in the remaining three phages (Table 2).

Phage polysaccharide depolymerases are important for infecting capsulated strains and biofilms. A search for depolymerases revealed that phages kpv33d1, kpv33d7, and kpv33s1 harbored two depolymerase sequences each, showing high homology to known depolymerases (Supplementary Table 5). Despite displaying the characteristic double-halo plaque morphology, no depolymerase sequences were identified in kpv33d4, and kpv33d6. In the case of kpvTh1, no depolymerase sequences were detected, consistent with its clean plaque morphology (Table 2).

The host range of lytic activity of selected phages was evaluated initially using the spot test assay (Supplementary Table 6). A high variability of infectivity spectrum was observed, although all phages were able to lyse at least three UTI strains, often belonging to different STs. Phages kpv33d1 and kpv33d4 showed the broadest activity, lysing 12 and 11 strains, respectively. Most susceptible isolates belonged to ST15 (6 and 5 strains, respectively), whereas only two ST147 isolates were susceptible in this assay. To confirm productive infection, double agar plaque assays were performed. A marked reduction in phage activity compared to the spot test was observed for all the phages. Notably, none of the phages were able to lyse ST15 strains in the double agar assay. Among the eight ST147 isolates, six were completely resistant, and only two were susceptible to kpv33d1, kpv33d4, and kpvTh1. Among the remaining STs, strain 2173 (ST392) was the most sensitive, being lysed by five phages. Strain 6022 (ST307) was lysed by three phages, and strain 5995 (ST17) by two phages (Supplementary Table 7).

To quantify phage lytic activity, the efficiency of plating (EOP) was calculated as the ratio between PFU in the target strain and PFU in the original host strain (Table 2). The analysis confirmed that strain 2173 (ST392, HMV) showed the highest susceptibility. Indeed, phages kpv33d1, kpv33d4, and kpv33d6 displayed higher activity against strain 2173 than against the original isolation host (KLEB-33).

**Figure 5:**
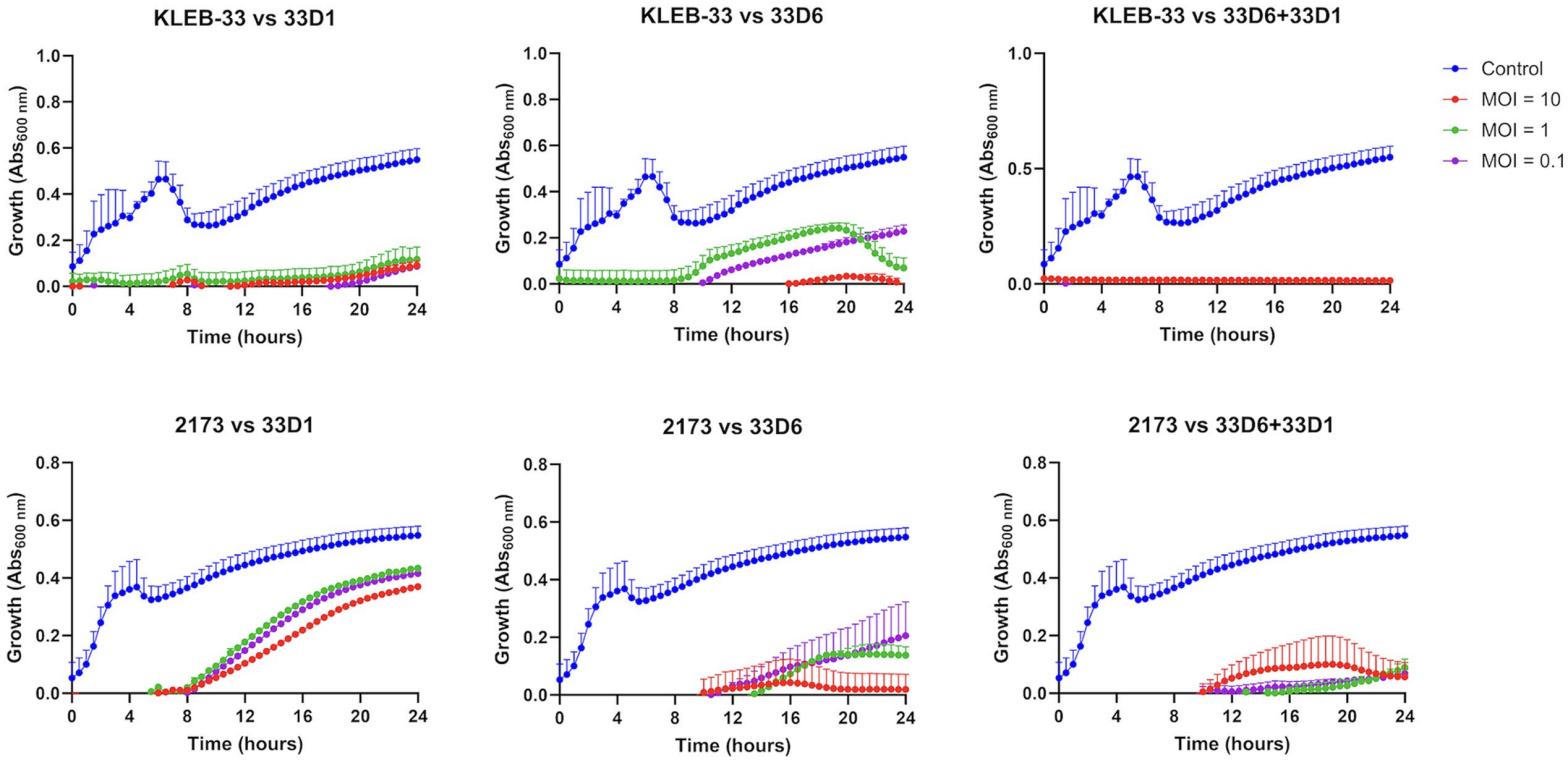
Growth curves of *K. pneumoniae* strain Kleb-33 (control) and the carbapenemase-producing UTI strain 2173 (ST392) challenged with phages *Webervirus* kpv33d1 and *Jiaodavirus* kpv33d6 in liquid cultures at multiplicity of infection (MOI) values 0.1, 1, and 10. Cultures (*n* = 3) were performed in 96-well microtiter plates incubated for 24 h at 37 °C, and growth was monitored every 30 min by measuring absorbance at 600 nm (Abs_600nm_).

For liquid infection assays, strain 2173 was selected due to its high susceptibility. Phages kpv33d1 (broad host range) and kpv33d6 (highly specific for strain 2173) were tested individually and in combination at MOIs of 0.1, 1 and 10. Results were compared with those obtained for KLEB-33. *Webervirus* kpv33d1 efficiently inhibited the planktonic growth of KLEB-33 at all MOIs (Figure 5). In strain 2173, it delayed growth for approximately 8 h, with limited differences between MOIs, but lost activity once the culture reached the stationary phase. *Jiaodavirus* kpv33d6 showed a similar pattern, although only MOI 10 effectively suppressed the growth of strain 2173. The combination of both phages resulted in stronger growth inhibition than either phage alone at all tested MOIs (Figure 5).

**Figure 6:**
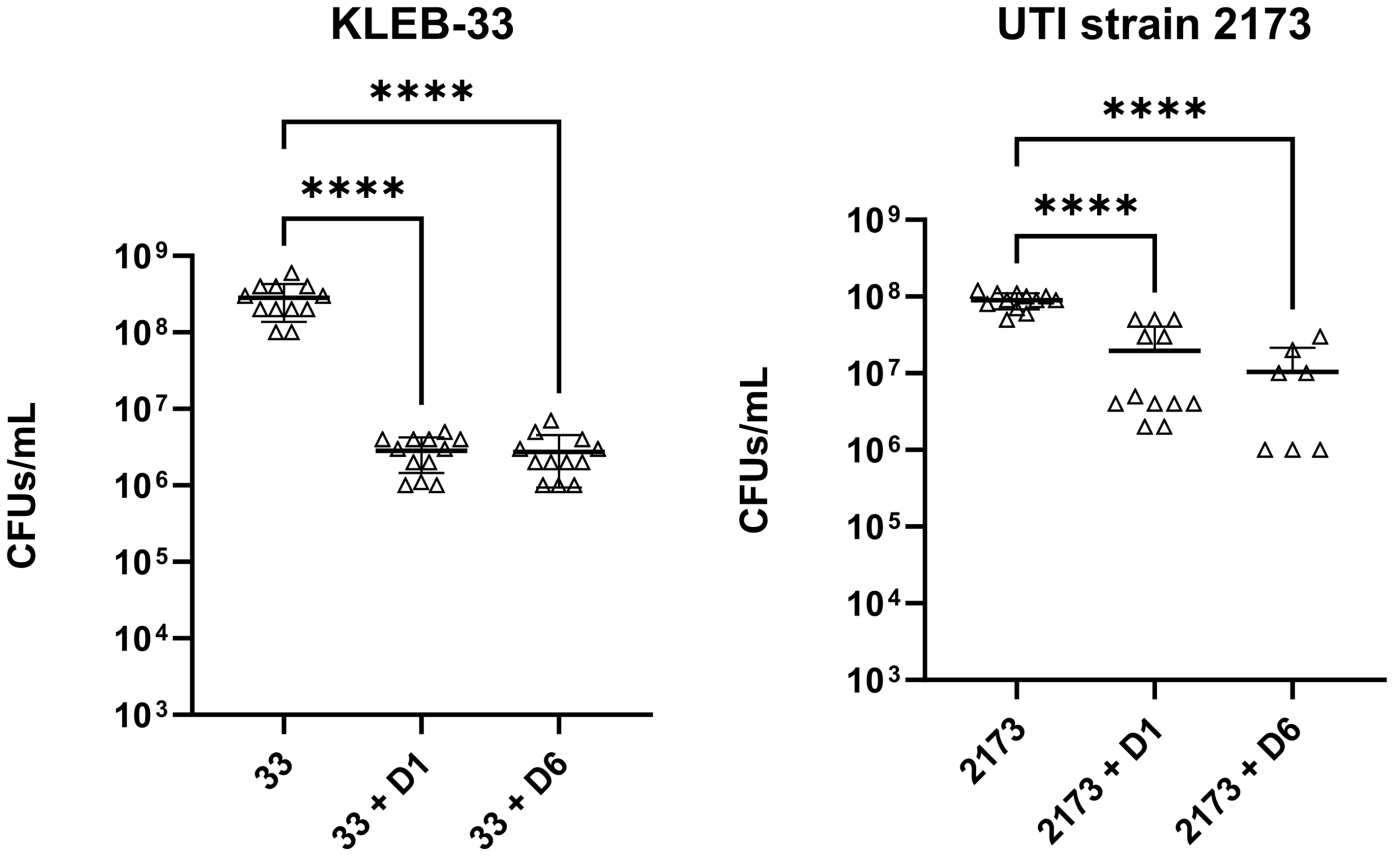
Lytic activity of *Webervirus* kpv33d1 and *Jiaodavirus* kpv33d6 on pre-formed biofilms of the carbapenemase-producing urine strain 2173 and the clinical, hyper-biofilm-forming model strain KLEB-33. Biofilms were cultivated for 24 h before being transferred to a 10^6^ PFU suspension of the phages (MOI 0.01). Remaining biofilm CFU counts were performed after 24 h. Stars indicate statistically significant differences of phage treatments in comparison with the untreated control (Student’s *t*-test, *p* < 0.001). ****: *p* < 0.00001 (Student’s t-test).

Furthermore, the activity of kpv33d1 and kpv33d6 (10^6^ PFU mL^−1^, MOI 0.01) was also evaluated against preformed biofilms of strain 2173, with biofilms of strain KLEB-33 also used as controls. Both phages significantly reduced CFU counts in biofilms (Student’s t-test, *p* < 0.001). Interestingly, strain KLEB-33, a strong biofilm former, was more susceptible than 2173, showing a reduction of two orders of magnitude, while a reduction close to 1 order of magnitude in viable cells was observed in the UTI strain 2173 biofilms (Figure 6).

## 4 Discussion

Urinary tract infections are among the most prevalent microbial diseases, and their economic impact on society is enormous. *K. pneumoniae* (Kpn) is the second leading cause of UTI. The ability of Kpn to evade the immune system, its increasing antimicrobial resistance, together with its capacity to deploy multiple virulence factors for colonization and dissemination, has turned this pathogen into a priority target for the development of strategies to prevent and control antimicrobial resistance, according to the WHO (Sati et al., 2025).

In this study, a collection of 24 carbapenemase-producing Kpn strains (CP-Kpn), most of them OXA-48, isolated from urinary tract infections between 2014 and 2022, was characterized in terms of antimicrobial resistance, resistance gene repertoire, virulence factors, hypermucoviscosity, and biofilm-forming ability. Phenotypic and genotypic characterization of Kpn is essential for the identification of UTI-associated traits and the development of alternative or adjuvant therapies to antibiotics, including phage therapy.

Carbapenem resistance is currently one of the most critical concerns among Kpn isolates worldwide (European Centre for Disease Prevention and Control and World Health Organization, 2023; World Health Organization, 2024; Sati et al., 2025). In Spain, the prevalence of CP-Kpn increased from 0.2% in 2009 (Miró et al., 2013) to 1.7% in 2013 (Oteo et al., 2015), and more recently, 2.5% was reported in a multicentre study (Cañada-García et al., 2022). In our collection of UTI-derived CP-Kpn isolates, the most frequent carbapenemase was OXA-48, detected in 23 of the 24 strains analyzed (95%). This finding is consistent with previous reports indicating that OXA-48 is the most prevalent carbapenemase gene in Spain, particularly in the northern region, with only sporadic detection of other carbapenemases (Oteo et al., 2015; Vázquez-Ucha et al., 2021). However, this percentage is higher than that reported in previous national studies, where the prevalence of OXA-48 among Kpn isolates was lower (69.8%; Cañada-García et al., 2022). Slightly higher values were reported in other nationwide studies conducted between 2014 and 2018 (73.5 and 82%; Vázquez-Ucha et al., 2021; Gracia-Ahufinger et al., 2023). In those studies, CP-Kpn isolates were recovered from different clinical sources, with urine samples accounting for 53.3% (418), 39.3% (215), and 56.6% (227) of the total samples, respectively. Dissemination of OXA-48 is mainly driven by the spread of a highly transferable IncL/M plasmid carrying *bla*_OXA-48_-like genes within the Tn1999 transposon (Poirel et al., 2012). In our study, the IncL plasmid was detected in 21 of the 24 strains, all harboring *bla*_OXA-48_, suggesting that horizontal transfer of this plasmid constitutes the principal dissemination mechanism.

High-risk AMR clones ST15/OXA-48 (11 strains), ST147/OXA-48 (8 strains), and ST307/OXA-48 (1 strain) were identified in our urine CP-Kpn collection. An increase in the frequency of these three clones has been observed in recent years (Cañada-García et al., 2022). In our urine isolates, ST distribution was not homogeneous over time: ST15 was detected in strains isolated up to 2016, whereas ST147 was recovered from 2015 through 2022 (Supplementary Table 2). Clone ST15 is a globally disseminated high-risk lineage responsible for the spread of extended-spectrum beta-lactamases (mainly CTX-M-15) and carbapenemases (Pitout et al., 2019). In Spain, ST15 accounted for approximately 13% of CP-Kpn isolates between 2013 and 2019, without significant temporal variation (Oteo et al., 2015; Cañada-García et al., 2022). ST307 and ST147 are also recognized as high-risk AMR clones worldwide and share several genomic characteristics. Our data indicate an increase in ST147 prevalence among urine isolates after 2016. This trend appears consistent with reports from other clinical sources, where ST147 was not among the most frequent clones in 2013 (Oteo et al., 2015) but ranked fifth in 2019 among isolates of diverse origin (Cañada-García et al., 2022). Only one ST307 isolate was identified in our urine collection, despite ST307 being the most prevalent clone nationally in 2019 (Cañada-García et al., 2022). These findings may suggest differences in the prevalence of high-risk clones in urinary infections compared with other infection sources, although analysis of a larger number of strains would be necessary to confirm this trend.

The most active antibiotics *in vitro* were colistin (91.6% susceptibility) and ceftazidime/avibactam (100% susceptibility). However, high resistance rates to other antibiotics were observed, with 83.3% of isolates classified as multidrug-resistant (MDR). Notable resistance to cephalosporins was detected, consistent with extended-spectrum beta-lactamase (ESBL) production. Genes encoding ESBLs were identified in 22 isolates, with *bla*_CTX-M-15_ being the most prevalent (16 isolates). The IncR plasmid, known to mediate dissemination of *bla*_CTX-M-15_ (Salazar et al., 2022), was detected in 17 strains, 13 of which carried *bla*_CTX-M-15_, suggesting preferential horizontal transfer of this gene via plasmid dissemination, similarly to *bla*_OXA-48_.

Aminoglycoside resistance genes were detected in 16 strains, including N-acetyltransferases and O-phosphotransferases. The 16S rRNA methyltransferase gene *rmtF* was detected in 7 strains, all belonging to ST147, consistent with previous reports identifying ST147 as the predominant lineage among OXA-48-producing Kpn strains carrying *rmtF* (Arca-Suárez et al., 2022). The *rmtF* enzyme confers high-level resistance to all aminoglycosides and was first described in 2012 in a Kpn isolate from Reunion Island (Galimand et al., 2012). Since then, it has been reported in multiple countries (Lee et al., 2014; Gamal et al., 2016; Caméléna et al., 2022), not only in ST147 but also in ST15 (Takei et al., 2024). Nevertheless, *rmtF* was not detected in any of our ST15 isolates.

Principal component analysis (PCA) showed clear clustering of strains into two main groups according to antibiotic resistance genes, corresponding to ST147 and ST15, and a similar separation was observed based on virulence genes (Figures 3A,C). However, phenotypic resistance profiles did not discriminate between STs (Figure 3B), illustrating phenotypic convergence of distinct genetic lineages under antibiotic selective pressure. When virulence genes were considered, ST307 clustered with ST147 (Figure 3C), supporting their close genetic relationship (Peirano et al., 2020), although this pattern was not observed when resistance genes were analyzed (Figure 3A), despite the presence of similar AMR determinants.

Kpn employs multiple strategies to adapt to its ecological niche and to protect itself against the host immune response. The hypermucoviscous (HMV) phenotype, type 1 and type 3 fimbriae, lipopolysaccharide (LPS), iron uptake and transport systems, toxins, and the ability to form biofilms are among the virulence factors involved in its pathogenesis (Paczosa and Mecsas, 2016). The HMV phenotype, determined by the string test, has been widely used as a marker for hypervirulent *K. pneumoniae* (hvKp), with a reported accuracy of approximately 90% for hvKp clinical strains (Russo et al., 2018). Indeed, the terms “HMV” and “hypervirulent” are often used interchangeably in the literature. Nevertheless, the high proportion of HMV strains observed in our urine Kpn collection (62.5%) adds to the growing evidence that these represent two distinct phenotypes and should not be considered synonymous (Catalán-Nájera et al., 2017; Choby et al., 2020), particularly since none of our strains were isolated from invasive infections or carried recognized hypervirulence biomarkers (Russo et al., 2018). Therefore, the string test should not be used as a definitive diagnostic tool for hypervirulence, especially in geographical areas with a low prevalence of hvKp. Notably, the proportion of HMV strains in our urine collection is substantially higher than that reported for clinical strains of diverse origin (10.5%) and for meat isolates (24%) analyzed under the same culture conditions (Silva-Bea et al., 2024b), which may suggest an adaptive advantage of the HMV phenotype for urinary tract colonization. Indeed, the HMV phenotype has been more often found in UTI isolates in comparison with those from healthy adults (Lin et al., 2010), and hospitalized patients infected with HMV-positive strains had a significantly higher risk of developing concurrent bacteraemia, highlighting the phenotype’s role in invasive disease (Kim et al., 2017). On the other hand, it has been demonstrated that both, hypervirulent and classical UTI isolates lose their HMV phenotype when grown in human urine (Khadka et al., 2023), and an hypomucoid variant of a CP-Kpn strain exhibited an increased residence time in the mouse urinary tract as well as a dramatic increase in adhesion to bladder epithelial cells and biofilm production, facilitating the colonization of the urinary tract (Song et al., 2024). In our CP-Kpn collection, the HMV phenotype does not correlate with biofilm formation *in vitro*. Moreover, the *rmpA* gene, which encodes a transcriptional regulator required for maximal capsule gene expression and confers hypermucoviscosity (Walker et al., 2020) and is often found in urine isolates(Lin et al., 2010), was not found in any of our strains (Table 1). The capsule and HMV-related genes *rmpD* and *rmpC* were also absent in our strains. Our assessment of the HMV phenotype, using two different culture media and microaerobic conditions, is more comprehensive than the standard string test method, and allows the detection of the HMV phenotype in a larger number of strains (Silva-Bea et al., 2024b). It should be noted that some of the strains showed the HMV phenotype only in the presence of glucose, indicating a complex regulatory network for this phenotype. These somehow contradictory results on the regulatory circuits involved in the HMV phenotype and its role in urinary tract infection require further investigation in order to elucidate the mechanisms underlying adherence and persistence of Kpn strains in the urinary tract.

The ability of Kpn to survive and proliferate in host tissues depends on the availability of essential nutrients, particularly iron, which is required for the synthesis of numerous enzymatic systems. In iron-limited environments such as the urinary tract, bacterial survival relies on siderophore-mediated iron acquisition (Holden and Bachman, 2015). All urine isolates harbored genes involved in enterobactin biosynthesis, including its receptor system (*fepABCDG*). This finding is consistent with previous reports: for example, the *entB* gene was detected in 97.5% of Kpn strains isolated from urine (Kao et al., 2023), and the enterobactin cluster was present in all isolates in a study in which 42.1% of strains were of urinary origin (Jati et al., 2023). Additionally, approximately 30% of Kpn strains causing UTI have been reported to carry yersiniabactin (Holt et al., 2015; Lam et al., 2018). In contrast, we observed a markedly higher prevalence in our collection: the complete *ybtAEPQSTUX* cluster was detected in 79.1% of strains, the complete *irp12* cluster was present in 70.8, and 87.5% carried the *fyuA* outer membrane receptor. These findings point to a potentially important role of the yersiniabactin system in uropathogenesis.

Aerobactin and salmochelin are less common in Kpn (<5% of isolates) and are typically plasmid-encoded virulence determinants (Lam et al., 2018). In our study, no genes involved in the biosynthesis of aerobactin or salmochelin were detected. However, the *iutA* gene, encoding the outer membrane receptor for aerobactin, was present in all isolates. This observation may indicate acquisition of receptor genes independently of the full biosynthetic cluster, or partial loss of an originally acquired operon, leaving only the receptor gene, as previously reported (Abdelwahab et al., 2021). In clinical isolates from Iran, *iutA* was found in approximately 46%, while *iucB* was present in only 24%, indicating many receptor strains without complete biosynthetic capability (Davoudabadi et al., 2023). The high prevalence of *iutA* among our UTI strains may suggest an adaptive advantage in urinary tract infection and warrants further investigation. Finally, the *kfuABC* iron acquisition cluster was detected in 54.1% of strains and was predominantly associated with ST15, in agreement with previous reports (Jati et al., 2023). Nine isolates lacked the complete cluster, while two of them lacked parts of the genes. Other virulence factors, such as the genotoxin colibactin and the biomarker *peg-344*, have been associated with increased virulence in certain *K. pneumoniae* lineages (Russo and Marr, 2019). However, none of the UTI CP-Kpn isolates in our study carried these determinants, suggesting that they do not appear to be required for the development of UTI.

Another relevant virulence factor is the periplasmic serine endoprotease DegP-like protein encoded by *htrA.* This protease plays an important role in protein quality control under stress conditions, contributing to bacterial survival in hostile environments such as the urinary tract. According to Kao et al. (2023), *htrA* is detected in most Kpn isolates from urine samples, suggesting a specific association with UTI. Our findings are consistent with this observation, as *htrA* was detected in all strains analyzed.

Biofilm formation is another important virulence-related trait. Recurrence of infection after antibiotic treatment may result from recolonization by bacteria surviving within biofilms. Moreover, biofilm-associated bacteria display increased resistance to antibiotics and phagocytosis compared to planktonic cells. The high biofilm-forming capacity of *K. pneumoniae* is well documented; however, the genetic basis underlying inter-strain variability remains unclear (Choby et al., 2020), and its precise role in pathogenesis is still debated, particularly since no significant differences were observed between food and clinical isolates in some studies (Silva-Bea et al., 2024b). In our study, 70.8% of urine CP-Kpn strains were classified as moderate or strong biofilm formers, and 37.5% were hyper-biofilm formers. This is in strong contrast with previous studies that reported that carbapenem resistant *K. pneumoniae* were less likely to be strong biofilm formers (Cusumano et al., 2019). This discrepancy could also be attributed to the biofilm culture system applied (Silva-Bea et al., 2024b). Considerable variability was observed among strains, independently of origin or HMV phenotype, although ST15 strains exhibited slight but significantly higher biofilm-forming capacity than ST147. Several studies have reported moderate or strong biofilm formation among urine isolates (Ballén et al., 2021), with percentages exceeding 80% in some reports (Vuotto et al., 2017; Li et al., 2022; Makhrmash et al., 2022). Furthermore, our urine CP-Kpn strains demonstrated stronger biofilm formation than Kpn clinical isolates of diverse origin cultivated under identical conditions, in which only 58% were intermediate or hyper-biofilm formers (Silva-Bea et al., 2024b). These findings may suggest that enhanced biofilm formation contributes to uropathogenicity. Nevertheless, such comparisons should be interpreted cautiously due to the limited sample size and the strong influence of experimental conditions on biofilm assessment, since *in vivo* biofilm forming capacity may change under the harassing conditions generated by the presence of urine, as discussed above for the HMV phenotype.

Different studies have linked biofilm formation to capsule type and/or fimbrial expression. The polysaccharide capsule is a key virulence factor that protects Kpn from phagocytosis, inhibits opsonization, and interferes with activation of the innate immune response (Paczosa and Mecsas, 2016). In our study, a strong correlation was observed between capsule locus (KL) type and sequence type: all KL112 isolates belonged to ST15 and all KL64 isolates to ST147. Kao et al. (2023) also reported KL64 as the most frequent capsule type among urine isolates.

Type 1 and type 3 fimbriae are major adhesins involved in the early stages of Kpn infection. Type 1 fimbrial genes are expressed in the urinary tract but not in the gastrointestinal tract or lungs, contributing to bladder cell invasion and biofilm formation during UTI (Paczosa and Mecsas, 2016). In our collection, genes encoding type 1 fimbriae (*fimABCDFGHI*) were detected in all but one strain (ST147 strain 1,604). Similarly high prevalence has been reported previously (Makhrmash et al., 2022), supporting their role in urinary colonization. Type 3 fimbriae facilitate biofilm formation on both living tissues and abiotic surfaces such as catheters (Paczosa and Mecsas, 2016). The *mrkABCDEHIJ* cluster was detected in all strains, consistent with previous observations in urine isolates (Mirzaie and Ranjbar, 2021), although other studies have reported lower frequencies (46.4–53.6%) in urine samples (Makhrmash et al., 2022).

The *kpiABCDEFG* operon encodes additional fimbrial adhesins associated with host colonization and biofilm formation and has been linked to the globally disseminated ST15 clone (Gato et al., 2020). In our study, the gene cluster was present in eight ST147 isolates and in the two isolates belonging to ST392 and ST307 (41.6% overall). A comparable frequency (33%) was reported in an OXA-48 Kpn collection of diverse origin (Jati et al., 2023). Notably, none of our ST15 isolates carried the kpi operon, highlighting lineage-specific variability.

Taken together, despite the high proportion of HMV strains (62.5%), the urinary origin of isolates, the high MDR rate (83.3%), and the absence of hypervirulence markers (*peg-344*, *iroB, iucA*, *rmpA*, *rmpA2*, aerobactin, and salmochelin; Russo et al., 2018; Chu et al., 2023; Lim et al., 2025), all strains in our study can be classified as classical Kpn (cKp). Although hvKp infections predominate in the Asia-Pacific region, 5.4% of Kpn isolates from bacteraemia episodes in Spain were reported as hvKp (Choby et al., 2020). Our results suggest that gene transfer between CP-Kpn and hvKp strains has not yet occurred in UTI isolates in our setting. Nevertheless, continuous genomic surveillance in clinical microbiology laboratories remains essential to detect early acquisition of hypervirulence genes by MDR cKp strains and to guide appropriate diagnosis and treatment strategies.

In this study, six Kpn bacteriophages (*Webervirus* kpv33d1, *Jiaodavirus* kpv33d4, *Jiaodavirus* kpv33d6, *Webervirus* kpv33d7, *Druslisvirus* kpv33s1, *Jiaodavirus* kpvth1) were isolated and characterized to evaluate their lytic activity against the 24 urine CP-Kpn strains. These phages were selected from a collection of 22 isolates obtained from urban and hospital wastewater using enrichment techniques with two genetically and phenotypically distinct host strains: the MDR, HMV, hyper-biofilm-forming clinical strain KLEB-33 (ST16, KL51) and the type strain ATCC 13883 T (ST3, KL3). Phages were selected based either on their capacity to lyse both model strains or on their high lytic capacity against their specific host strain. Both strains are capsulated, although the capsule is thicker in the type strain (Silva-Bea et al., 2024a). Genome sequencing revealed the absence of lysogeny-related genes, antibiotic resistance genes, and virulence factors, supporting their suitability for phage therapy (Pirnay et al., 2024). CRISPR-Cas and restriction-modification (RM) systems are detected in most CP-Kpn strains, in agreement with previous reports (Orzechowska and Mohammed, 2019; Costa et al., 2024). However, anti-CRISPR systems were not identified in any phage genome, and anti-RM systems were detected in only one phage (Table 2).

All six phages lysed at least one of the 24 UTI CP-Kpn strains. Phages kpv33d1, kpv33d4, and kpv33d6 exhibited higher efficiency of plating (EOP) against strain 2173 (ST392, KL27) than against their original host KLEB-33 (Table 2). Three phages (*Webervirus* kpv33d1, *Jiaodavirus* kpv33d4, and *Jiaodavirus* kpvth1) lysed strains belonging to different STs and KL types. Although polysaccharide depolymerases may contribute to broader host range, lytic activity was not strictly dependent on detectable depolymerase genes. For example, *Jiaodavirus* kpvth1 formed clear plaques without translucent halos, suggesting the absence of polysaccharide depolymerase activity (Table 2). Conversely, halos were observed for *Jiaodavirus* kpv33d4 and kpv33d6, despite the absence of identifiable depolymerase homologs in their genomes.

None of the phages lysed ST15 strains in the double agar assay, confirming previously reported specificity linked to ST and KL types (Kondo et al., 2023; Lourenço et al., 2023). Consistently, none of the phages was able to lyse the closely related strains 2539 (ST1961) or 2465 (ST449). Nevertheless, taxonomic proximity does not always predict susceptibility, as phages isolated using capsulated host strains belonging to ST16 and ST3 were capable of lysing strains of different STs and KL types. This finding contrasts with reports suggesting a broader host range when phages are isolated using non-capsulated strains (Lourenço et al., 2023). Moreover, previous results reported that both, resistant and susceptible isolates to a cocktail of 12 phages could be identified among strains with the same ST and KL type, including ST15 isolates (Ferriol-González et al., 2024). It should be noted that the tested phages have been isolated using only 2 host strains and therefore, future strategies of phage isolation should include more host strains, presenting a diverse genomic background and phenotypic traits in order to ensure the effectiveness of phage therapy against a wide range of pathogenic strains.

*Jiaodavirus* kpvth1 and *Jiaodavirus* kpv33d6 share 99% genomic identity but display distinct plaque morphologies and host ranges. They differ by three nucleotide substitutions in a gene encoding a protein homologous to A0A1B1P8Y2, containing two predicted transmembrane *α*-helices. Minor genomic differences have previously been shown to influence host range (Lourenço et al., 2023).

Consistent with previous reports (Khan Mirzaei and Nilsson, 2015), spot assays yielded more positive results than double-layer plaque assays. The loss of activity affected all the high-risk strains belonging to ST15. Although this discrepancy could be attributed to lysis from without (Abedon, 2011; Khan Mirzaei and Nilsson, 2015) or residual lytic enzymes, spot-test results were not reproducible after phage propagation from frozen stocks. Although good stability of *Staphylococcus* phages in glycerol at −80 °C has been described (González-Menéndez et al., 2018), the observed loss of activity warrants further investigation and may be related to structural modifications of phage receptor-binding proteins during storage, which may be specific to strains in ST15.

Two phages were further evaluated in liquid cultures and biofilm assays against strain 2173 (ST392, KL27). *Webervirus* kpv33d1 (broad host range) and *Jiaodavirus* kpv33d6 (highly productive but narrow host range) were selected. Despite regrowth being observed in both, KLEB-33 and strain 2173 when phages were applied individually, growth remained lower than untreated controls, and regrowth was MOI-dependent. Emergence of resistant mutants has been widely reported and is not always prevented by phage cocktails (Kondo et al., 2023; Le et al., 2023; Lourenço et al., 2023; Chen et al., 2024). In our study, the combined application of both phages markedly reduced resistance development, even at low MOI (0.01) and long-lime incubation (24 h), supporting the selection of complementary phages for cocktail design. Both phages also reduced bacterial load in preformed biofilms. Although eradication was not achieved, the low MOI applied may have limited maximal activity. Selection of biofilm-targeting phages or adjunctive strategies, such as depolymerase application, may improve therapeutic efficacy.

Although capsule loss has been described as an adaptive advantage in urinary infections (Ernst et al., 2020), all strains in our CP-Kpn urine collection presented the *rcsABCDF* cluster and most of them were hypermucoviscous (Table 1). Since capsule composition influences phage susceptibility, evaluating capsule phase variation under different environmental conditions would be relevant. For the clinical application of phage therapy in the treatment of Kpn UTI, it is therefore necessary to expand our knowledge of the *in vivo* physiology of Kpn in the urinary tract, since important traits affecting phage susceptibility, such as capsule, HMV and other adherence-related traits, may change in the urinary environment (Song et al., 2024). A combined strategy employing phages isolated against both capsulated and non-capsulated strains may therefore be advisable. In this sense, the selection of bacteriophages with wide-spectrum depolymerase activity may be an interesting strategy to widen the lytic spectrum (Zurabov et al., 2023). The use of mixed strategies, such as adding wide-spectrum depolymerases or other lytic enzymes to phage cocktail formulations, could be another effective approach for Kpn treatment and should also be explored (Islam et al., 2024).

Overall, several virulence traits appear enriched among urine CP-Kpn isolates, including high prevalence of the yersiniabactin cluster, universal presence of type 3 fimbriae and *htrA,* increased biofilm-forming capacity, and high frequency of the HMV phenotype. Additionally, the *kpiABCDEFG* cluster was associated with ST147 rather than ST15 in our collection. The contribution of these traits to urinary tract colonization should be confirmed in larger cohorts and mechanistic studies. Finally, our findings demonstrate the feasibility of isolating lytic bacteriophages active against capsulated UTI-derived Kpn strains. Although resistance development occurred, combined phage application effectively suppressed planktonic growth even at low MOI. The potential contribution of depolymerase activity to biofilm degradation warrants further investigation.

## Data Availability

The datasets presented in this study can be found in online repositories. The names of the repository/repositories and accession number(s) can be found at: https://www.ncbi.nlm.nih.gov/genbank/, JBWJIH000000000-JBWJJE000000000; https://www.ncbi.nlm.nih.gov/genbank/, submission IDs: 3068750, 3,069,748, 30,697,754, 3,069,774, 3,069,833, and 3,069,848.

## Footnotes

1
https://pathogen.watch

2
https://bigsdb.pasteur.fr/klebsiella/

3
https://www.cgmlst.org/ncs/schema/Kpneumoniae4113/

4
https://microreact.org/

5
https://cge.food.dtu.dk/services/ResFinder/

6
https://card.mcmaster.ca

7
https://pathogen.watch
