# Supplementary material for: Genomic and virulence characterization of OXA-48-producing Klebsiella pneumoniae causing urinary tract infections and their susceptibility to lytic bacteriophages
Source: Front Microbiol. 2026 Jun 9;17:1849761. doi: 10.3389/fmicb.2026.1849761 (PMC13286925; doi:10.3389/fmicb.2026.1849761)
Supplement: Supplementary file 1 [file Data_Sheet_1.pdf]

# Genomic and virulence characterization of OXA-48-producing *Klebsiella pneumoniae* causing urinary tract infections and their susceptibility to lytic bacteriophages

**Sonia Rey, Sergio Silva-Bea, Carlos Davina-Nunez, Manuel Romero, Belén Fontán-Silva,**

**Sonia Pérez, Ana Otero**

***Supplementary Material***

## Supplementary Table 1

**Table 1S**. *K. pneumoniae* virulence genes searched in this study

| Phenotype | Gene Name | Sources | Function | References |
|---|---|---|---|---|
| **Enterobactin siderophore** | *entA* | NCBI | dihydroxymethyladenine dehydrogenase (*entA)* | 1-6 |
| | *entB* | NCBI | isochorismatase (*entB)* | 1-6 |
| | *entC* | NCBI | isochorismate synthase (*entC)* | 1-6 |
| | *entD* | NCBI | 4'-phosphopantetheinyl transferase (*entD*) | 1-6 |
| | *entE* | Pasteur DB | 2,3-dihydroxybenzoate-AMP_ligase (*entE*) | 1-6 |
| | *entF* | Pasteur DB | enterobactin synthase subunit F (*entF*) | 1-6 |
| | *fepA* | NCBI | ferrienterobactin outer membrane transporter | 2,4,6 |
| | *fepB* | NCBI | ferrienterobactin ABC transporter periplasmic binding protein | 2,4,6 |
| | fepC | NCBI | ferrienterobactin ABC transporter ATPase | 2,4,6 |

| | | | | |
|---|---|---|---|---|
| | *fepD* | NCBI | ferrienterobactin ABC transporter permease | 2,4,6 |
| | *fepG* | NCBI | iron-enterobactin ABC transporter permease | 2,4,6 |
| **Salmochelin siderophore** | *iroB* | Pasteur DB | Salmochelin siderophore glycosyltransferase | 1-5, 7-9 |
| | *iroC* | Pasteur DB | salmochelin transporter | 1-5, 7-9 |
| | *iroD* | Pasteur DB | salmochelin degradation | 1-5, 7-9 |
| | *iroN* | Pasteur DB | catecholate siderophore receptors | 1-5, 7-9 |
| **Aerobactin siderophore** | *iucA* | Pasteur DB | aerobactin synthetase I | 1,2,4,5,7,9 |
| | *iucB* | Pasteur DB | N6 -hydroxy-L-lysine (hLys) acetyltransferase creating aerobactin precursor (cleavable chaperone chimera) | 1,2,4,5,7,9 |
| | *iucC* | Pasteur DB | aerobactin synthetase II | 1,2,4,5,7,9 |
| | *iucD* | Pasteur DB | hydroxylating L-lysine | 1,2,4,5,7,9 |
| | *iutA* | Pasteur DB | aerobactin siderophore receptor | 2-5,7,8 |
| **Yersiniabactin siderophore** | *ybtA* | Pasteur DB | *Ybt* transcriptional regulator | 1,3-5,10 |
| | *ybtE* | Pasteur DB | 2,3 dihydrobenzoate AMP ligase | 1,3-5,10 |
| | *ybtP* | Pasteur DB | ATPase/ABC transporter protein | 1,3-5,10 |
| | *ybtQ* | Pasteur DB | involved in Fe-Ybt transport | 1,3-5,10 |
| | *ybtT* | Pasteur DB | thioesterase enzyme | 1,3-5,10 |
| | *ybtU* | Pasteur DB | thiazolinyl imide reductase | 1,3-5,10 |
| | *ybtS* | Pasteur DB | Salicylate synthetase | 1,3-5,10 |

| | | | | |
|---|---|---|---|---|
| | *ybtX* | Pasteur DB | major facilitator superfamily protein | 1,3-5,10 |
| | *irp2* | Pasteur DB | iron acquisition yersiniabactin synthesis enzyme | 4,5,9,10 |
| | *irp1* | Pasteur DB | polyketide synthetase | 4,5,9,10 |
| | *fyuA* | Pasteur DB | receptor for uptake of Fe-Ybt | 4,5,10 |
| **Genotoxin: colibactin** | *clbA* | Pasteur DB | acessory protein: phosphopantetheinyl transferase activate other enzymes | 1,3,4,11 |
| | *clbB* | Pasteur DB | synthesis enzyme : NRPS-PKS | 1,3,4,11 |
| | *clbC* | Pasteur DB | synthesis enzyme : PKS | 1,3,4,11 |
| | *clbD* | Pasteur DB | synthesis enzyme : hydroxyacyl-coa dehydrogenase | 1,3,4,11 |
| | *clbE* | Pasteur DB | synthesis enzyme : acyl carrier protein | 1,3,4,11 |
| | *clbF* | Pasteur DB | synthesis enzyme : alfabeta dehydrogenase | 1,3,4,11 |
| | *clbG* | Pasteur DB | synthesis enzyme : acyl transferase | 1,3,4,11 |
| | *clbH* | Pasteur DB | synthesis enzyme : NRPS | 1,3,4,11 |
| | *clbI* | Pasteur DB | synthesis enzyme : PKS | 1,3,4,11 |
| | *clbL* | Pasteur DB | synthesis enzyme : amidase | 1,3,4,11 |
| | *clbM* | Pasteur DB | MATE transporter, transport precolibactin across cytoplasmic membrane | 1,3,4,11 |
| | *clbN* | Pasteur DB | synthesis enzyme : NRPS | 1,3,4,11 |
| | *clbO* | Pasteur DB | synthesis enzyme : PKS | 1,3,4,11 |
| | *clbP* | Pasteur DB | fmtA peptidase, transport precolibactin | 1,3,4,11 |

| | | | | |
|---|---|---|---|---|
| | *clbQ* | Pasteur DB | synthesis enzyme : thioesterase | 1,3,4,11 |
| | *clbR* | Pasteur DB | putative transcriptional regulatory protein | 1,3,4,11 |
| | *clbJ* | Pasteur DB) | synthesis enzyme : NRPS | 1,3,4,11 |
| | *clbK* | Pasteur DB | synthesis enzyme : PKS | 1,3,4,11 |
| **Type1 Fimbriae** | *fimA* | NCBI | "major structural gene,Fimbrial protein type 1, | 2,4,12-14 |
| | *fimB* | NCBI | regulator ON | 2,4,12-14 |
| | *fimC* | NCBI | molecular chaperone FimC | 2,4,12-14 |
| | *fimD* | NCBI | Usher protein | 2,4,12-14 |
| | *fimE* | NCBI | regulator OFF | 2,4,12-14 |
| | *fimF* | NCBI | minor subunit | 2,4,12-14 |
| | *fimG* | NCBI | minor subunit | 2,4,12-14 |
| | *fimH* | NCBI | type 1 fimbriae adhesin | 2,4,12-14 |
| | *fimI* | NCBI | fimbrial protein | 2,4,12-14 |
| **Type3 Fimbriae** | *mrkA* | Pasteur DB | major structural component; also adheres to abiotic surfaces in biofilms | 2,4,5,13-15 |
| | *mrkB* | Pasteur DB | Chaperone | 2,4,5,13-15 |
| | *mrkC* | Pasteur DB | Usher protein | 2,4,5,13-15 |
| | *mrkD* | Pasteur DB | tip adhesin | 2,4,5,13-15 |
| | *mrkF* | Pasteur DB | type 3 fimbriae anchor protein | 2,4,5,13-15 |
| | *mrkH* | Pasteur DB | novel c-di-GMP-dependent transcriptional activator | 2,4,5,13-15 |

| | | | | |
|---|---|---|---|---|
| | *mrkI* | Pasteur DB | transcriptional regulator | 2,4,5,13-15 |
| | *mrkJ* | Pasteur DB | phosphodiesterase | 2,4,5,13-15 |
| **Allantoin Metabolism** | *allS* | Pasteur DB | transcriptional activator AllS, activator of the allDC-ylbA operon involved in allantoin utilization, | 3-5,14,15 |
| | *allA* | Pasteur DB | Ureidoglycolate lyase | 3-5,14,15 |
| | *allB* | Pasteur DB | allantoinase: converse allantoin into allantoate | 3-5,14,15 |
| | *allC* | Pasteur DB | allantoate amidohydrolase: produces S-ureidoglycolate to oxalurate | 3-5,14,15 |
| | *allD* | Pasteur DB | ureidoglycolate dehydrogenase | 3-5,14,15 |
| | *allR* | Pasteur DB | repressor that controls regulon expression, modulated by glyoxylate | 3-5,14,15 |
| | *ybbW* | Pasteur DB | Allantoin permease | 5 |
| **Iron uptake** | *kfuABC* | Pasteur DB | A mediator of ferric 3+ Iron uptake | 1-3,5,14,15 |
| **Mucoviscosity** | *rmpA* | Pasteur DB | Mucoviscosity phenotype regulator protein | 1,3,4,5,8,9, 14,15 |
| | *rmpA2* | Pasteur DB | Mucoviscosity phenotype regulator protein | 1,3,4,5,8,9, 14,15 |
| **Fimbrial adhesin** | *kpiA* | NCBI | fimbrial protein | 16 |
| | *kpiB* | NCBI | molecular chaperone | 16 |

| | | | | |
|---|---|---|---|---|
| | *kpiC* | NCBI | fimbrial protein | 16 |
| | *kpiD* | NCBI | fimbrial protein | 16 |
| | *kpiE* | NCBI | molecular chaperone | 16 |
| | *kpiF* | NCBI | molecular chaperone | 16 |
| | *kpiG* | NCBI | fimbrial biogenesis outer membrane usher protein | 16 |
| - | *peg-344* | NCBI | A metabolite transporter of unknown function | 1,4,8,9,17 |
| **Tellurite resistance** | *terABCDE WXYZ* | NCBI | Confers resistance to tellurite | 1,4,9 |
| **Capsule synthesis** | *rcsABCDF* | NCBI | Capsule synthesis regulator | 2,4 |
| | *kvgAS* | Pasteur DB | Capsula polysaccharide synthesis reguator | - |
| **Microcin** | *mceABCD EGHIJ* | Pasteur DB | maduration microcin | 5 |
| **avoid stress** | *htrA* | NCBI | Periplasmic serine endoprotease DegP-like | 18,19,20 |

- **References**


1. Russo TA, Marr CM. Hypervirulent *Klebsiella pneumoniae*. Clin Microbiol Rev. 2019 May 15;32(3):e00001-19. doi: 10.1128/CMR.00001-19. PMID: 31092506; PMCID: PMC6589860.
2. Zhu J, Wang T, Chen L, Du H. Virulence Factors in Hypervirulent *Klebsiella pneumoniae*. Front Microbiol. 2021 Apr 8;12:642484. doi: 10.3389/fmicb.2021.642484. PMID: 33897652; PMCID: PMC8060575.
3. Choby JE, Howard-Anderson J, Weiss DS. Hypervirulent *Klebsiella pneumoniae* - clinical and molecular perspectives. J Intern Med. 2020 Mar;287(3):283-300. doi: 10.1111/joim.13007. Epub 2019 Nov 21. PMID: 31677303; PMCID: PMC7057273.
4. Dai P, Hu D. The making of hypervirulent *Klebsiella pneumoniae*. J Clin Lab Anal. 2022 Dec;36(12):e24743. doi: 10.1002/jcla.24743. Epub 2022 Nov 8. PMID: 36347819; PMCID: PMC9757020.
5. Shankar C, Veeraraghavan B, Nabarro LEB, Ravi R, Ragupathi NKD, Rupali P. Whole genome analysis of hypervirulent *Klebsiella pneumoniae* isolates from community and hospital acquired bloodstream infection. BMC Microbiol. 2018 Jan 8;18(1):6. doi: 10.1186/s12866-017-1148-6. PMID: 29433440; PMCID: PMC5809863.
6. Raymond KN, Dertz EA, Kim SS. Enterobactin: an archetype for microbial iron transport. Proc Natl Acad Sci U S A. 2003 Apr 1;100(7):3584-8. doi: 10.1073/pnas.0630018100. Epub 2003 Mar 24. PMID: 12655062; PMCID: PMC152965.
7. Bialek-Davenet S, Criscuolo A, Ailloud F, Passet V, Jones L, Delannoy-Vieillard AS, Garin B, Le Hello S, Arlet G, Nicolas-Chanoine MH, Decré D, Brisse S. Genomic definition of hypervirulent and multidrug-resistant *Klebsiella pneumoniae* clonal groups. Emerg Infect Dis. 2014 Nov;20(11):1812-20. doi: 10.3201/eid2011.140206. PMID: 25341126; PMCID: PMC4214299.
8. Li J, Li Y, Tang M, Xia F, Min C, Hu Y, Wang H, Zhang J, Zou M. Distribution, characterization, and antibiotic resistance of hypervirulent *Klebsiella pneumoniae* isolates in a Chinese population with asymptomatic bacteriuria. BMC Microbiol. 2022 Jan 18;22(1):29. doi: 10.1186/s12866-021-02413-w. PMID: 35042478; PMCID: PMC8764819.
9. Russo TA, Olson R, Fang CT, Stoesser N, Miller M, MacDonald U, Hutson A, Barker JH, La Hoz RM, Johnson JR. Identification of Biomarkers for Differentiation of Hypervirulent *Klebsiella pneumoniae* from Classical *K. pneumoniae*. J Clin Microbiol. 2018 Aug 27;56(9):e00776-18. doi: 10.1128/JCM.00776-18. PMID: 29925642; PMCID: PMC6113484.
10. Lam MMC, Wick RR, Wyres KL, Gorrie CL, Judd LM, Jenney AWJ, Brisse S, Holt KE. Genetic diversity, mobilisation and spread of the yersiniabactin-encoding mobile element ICEKp in *Klebsiella pneumoniae* populations. Microb Genom. 2018 Sep;4(9):e000196. doi: 10.1099/mgen.0.000196. Epub 2018 Jul 9. PMID: 29985125; PMCID: PMC6202445.
11. Faïs T, Delmas J, Barnich N, Bonnet R, Dalmasso G. Colibactin: More Than a New Bacterial Toxin. Toxins (Basel). 2018 Apr 10;10(4):151. doi: 10.3390/toxins10040151. PMID: 29642622; PMCID: PMC5923317.
12. Struve C, Bojer M, Krogfelt KA. Characterization of *Klebsiella pneumoniae* type 1 fimbriae by detection of phase variation during colonization and infection and impact on virulence. Infect Immun. 2008 Sep;76(9):4055-65. doi: 10.1128/IAI.00494-08. Epub 2008 Jun 16. PMID: 18559432; PMCID: PMC2519443.
13. Alcántar-Curiel MD, Ledezma-Escalante CA, Jarillo-Quijada MD, Gayosso-Vázquez C, Morfín-Otero R, Rodríguez-Noriega E, Cedillo-Ramírez ML, Santos-Preciado JI, Girón JA. Association of Antibiotic Resistance, Cell Adherence, and Biofilm Production with the Endemicity of Nosocomial *Klebsiella pneumoniae*. Biomed Res Int. 2018 Sep 23;2018:7012958. doi: 10.1155/2018/7012958. PMID: 30345305; PMCID: PMC6174813.
14. Catalán-Nájera JC, Garza-Ramos U, Barrios-Camacho H. Hypervirulence and hypermucoviscosity: Two different but complementary *Klebsiella* spp. phenotypes? Virulence. 2017 Oct 3;8(7):1111-1123. doi: 10.1080/21505594.2017.1317412. Epub 2017 Apr 12. PMID: 28402698; PMCID: PMC5711391.
15. Bialek-Davenet S, Criscuolo A, Ailloud F, Passet V, Jones L, Delannoy-Vieillard AS, Garin B, Le Hello S, Arlet G, Nicolas-Chanoine MH, Decré D, Brisse S. Genomic definition of hypervirulent and multidrug-resistant *Klebsiella pneumoniae* clonal groups. Emerg Infect Dis. 2014 Nov;20(11):1812-20. doi: 10.3201/eid2011.140206. PMID: 25341126; PMCID: PMC4214299.
16. Gato E, Vázquez-Ucha JC, Rumbo-Feal S, Álvarez-Fraga L, Vallejo JA, Martínez-Guitián M, Beceiro A, Ramos Vivas J, Sola Campoy PJ, Pérez-Vázquez M, Oteo Iglesias J, Rodiño-Janeiro BK, Romero A, Poza M, Bou G, Pérez A. Kpi, a chaperone-usher pili system associated with the worldwide-disseminated high-risk clone *Klebsiella pneumoniae* ST-15. Proc Natl Acad Sci U S A. 2020 Jul 21;117(29):17249-17259. doi: 10.1073/pnas.1921393117. Epub 2020 Jul 8. PMID: 32641516; PMCID: PMC7382220.
17. Bulger J, MacDonald U, Olson R, Beanan J, Russo TA. Metabolite Transporter PEG344 Is Required for Full Virulence of Hypervirulent *Klebsiella pneumoniae* Strain hvKP1 after Pulmonary but Not Subcutaneous

Challenge. Infect Immun. 2017 Sep 20;85(10):e00093-17. doi: 10.1128/IAI.00093-17. PMID: 28717029; PMCID: PMC5607406.
18. Mirzaie A, Ranjbar R. Antibiotic resistance, virulence-associated genes analysis and molecular typing of *Klebsiella pneumoniae* strains recovered from clinical samples. AMB Express. 2021 Aug 30;11(1):122. doi: 10.1186/s13568-021-01282-w. PMID: 34460016; PMCID: PMC8405773.
19. Cortés G, de Astorza B, Benedí VJ, Albertí S. Role of the htrA gene in *Klebsiella pneumoniae* virulence. Infect Immun. 2002 Sep;70(9):4772-6. doi: 10.1128/IAI.70.9.4772-4776.2002. PMID: 12183518; PMCID: PMC128236.
20. Xue RY, Liu C, Xiao QT, Sun S, Zou QM, Li HB. HtrA family proteases of bacterial pathogens: pros and cons for their therapeutic use. Clin Microbiol Infect. 2021 Apr;27(4):559-564. doi: 10.1016/j.cmi.2020.12.017. Epub 2021 Jan 5. PMID: 33359376.

**Supplementary Table 2**. Date of isolation, origin (nosocomial, community-acquired), type of urine sample (midstream, indwelling catheter and nephrostomy), ST and antibiotic resistance profile (S: sensitive, R: resistant, I: intermediate, according to EUCAST v13.0 clinical breakpoints for Enterobacterales) of the 24 CP-Kpn urine strains studied.

| STRAIN ID | DATE | ORIGIN | COLLECTION PROCEDURE | ST | AMP | AUG | PTZ | CXM | CTX | CAZ | FEP | CAZ-AVI | ETP | IP | MP | GM | TOB | AK | CIP | SXT | NIT | FOS | CT |
|---|---|---|---|---|---|---|---|---|---|---|---|---|---|---|---|---|---|---|---|---|---|---|---|
| 1589 | 10/06/2014 | NOSOCOMIAL | MIDSTREAM | 15 | R | R | R | R | R | R | R | S | R | S | S | S | R | S | R | I | S | R | S |
| 1681 | 11/07/2014 | NOSOCOMIAL | MIDSTREAM | 15 | R | R | R | S | S | S | S | S | R | S | S | S | S | S | R | S | S | S | S |
| 1665 | 09/10/2014 | NOSOCOMIAL | MIDSTREAM | 15 | R | R | R | R | R | R | R | S | R | I | S | S | R | S | R | R | S | R | S |
| 1674 | 19/11/2014 | COMMUNITY | MIDSTREAM | 15 | R | R | R | R | R | S | I | S | R | R | I | S | S | S | R | R | S | R | S |
| 1890 | 15/05/2015 | NOSOCOMIAL | MIDSTREAM | 15 | R | R | R | R | R | S | S | S | R | I | S | S | S | S | R | S | R | S | S |
| 1892 | 26/05/2015 | NOSOCOMIAL | MIDSTREAM | 15 | R | R | R | S | S | S | S | S | R | I | S | R | R | S | R | R | S | R | S |
| 1898 | 15/07/2015 | NOSOCOMIAL | MIDSTREAM | 15 | R | R | R | R | S | S | S | S | R | S | S | S | S | S | R | S | S | S | S |
| 1899 | 17/07/2015 | COMMUNITY | INDWELLING CATHETER | 15 | R | R | R | R | R | R | R | S | R | R | R | S | R | S | R | R | S | R | S |
| 1907 | 04/09/2015 | NOSOCOMIAL | MIDSTREAM | 15 | R | R | R | R | R | R | R | S | R | R | R | S | R | S | R | R | R | R | S |
| 1944 | 30/09/2015 | COMMUNITY | MIDSTREAM | 15 | R | R | R | R | S | S | S | S | R | S | S | S | S | S | R | S | S | R | S |
| 1954 | 16/11/2015 | NOSOCOMIAL | MIDSTREAM | 147 | R | R | R | R | R | R | R | S | R | R | R | R | R | R | R | R | R | S | S |
| 1925 | 19/02/2016 | NOSOCOMIAL | MIDSTREAM | 147 | R | R | R | R | R | R | R | S | R | R | R | R | R | R | R | R | R | S | S |
| 1997 | 05/03/2016 | NOSOCOMIAL | MIDSTREAM | 147 | R | R | R | R | R | R | R | S | R | R | R | R | R | R | R | R | R | S | R |
| 2096 | 21/06/2016 | NOSOCOMIAL | INDWELLING CATHETER | 147 | R | R | R | R | R | R | R | S | R | R | R | R | R | R | R | R | R | S | S |
| 2100 | 07/07/2016 | COMMUNITY | MIDSTREAM | 15 | R | R | R | R | R | R | R | S | R | I | S | S | S | S | R | S | S | R | S |
| 2144 | 12/09/2016 | NOSOCOMIAL | MIDSTREAM | 147 | R | R | R | R | R | R | R | S | R | R | R | R | R | R | R | R | R | S | R |
| 2173 | 20/01/2017 | NOSOCOMIAL | NEPHROSTOMY | 392 | R | R | R | R | R | R | R | S | R | R | R | R | R | S | R | R | R | R | S |
| 2343 | 26/09/2017 | NOSOCOMIAL | MIDSTREAM | 147 | R | R | R | R | R | R | R | S | R | R | R | R | R | R | R | R | R | R | S |
| 2539 | 11/04/2018 | COMMUNITY | MIDSTREAM | 1961 | R | R | R | R | R | R | R | S | R | R | R | S | S | S | R | S | S | R | S |
| 2465 | 21/12/2018 | NOSOCOMIAL | MIDSTREAM | 449 | R | R | R | S | S | S | S | S | R | S | S | S | S | S | S | S | S | S | S |
| 5995 | 17/08/2022 | NOSOCOMIAL | INDWELLING CATHETER | 17 | R | R | R | R | R | R | R | S | R | S | S | R | R | S | S | S | S | S | S |
| 6022 | 23/08/2022 | COMMUNITY | INDWELLING CATHETER | 307 | R | R | R | R | R | R | R | S | R | S | S | R | R | S | R | R | S | S | S |
| 6106 | 06/09/2022 | COMMUNITY | MIDSTREAM | 147 | R | R | R | R | R | R | R | S | R | R | R | R | R | R | R | S | R | R | S |
| 3503 | 24/10/2022 | NOSOCOMIAL | INDWELLING CATHETER | 147 | R | R | R | R | R | R | R | S | R | S | S | R | R | S | R | R | R | R | S |

**Supplementary Table 3**. Plasmids found in the 24 carbapenem-resistant *Klebsiella pneumoniae* strains isolated from urinary tract infection samples used in this study. Presence: grey color, absence: white.

| ID strain | | 1589 | 1665 | 1674 | 1681 | 1890 | 1892 | 1898 | 1899 | 1907 | 1944 | 2100 | 5995 | 1925 | 1954 | 1997 | 2096 | 2144 | 2343 | 6106 | 3503 | 6022 | 2173 | 2465 | 2539 |
|---|---|---|---|---|---|---|---|---|---|---|---|---|---|---|---|---|---|---|---|---|---|---|---|---|---|
| MLST | | 15 | 15 | 15 | 15 | 15 | 15 | 15 | 15 | 15 | 15 | 15 | 17 | 147 | 147 | 147 | 147 | 147 | 147 | 147 | 147 | 307 | 392 | 449 | 1961 |
| PLASMIDS | IncL | | | | | | | | | | | | | | | | | | | | | | | | |
| | IncFIB(K) | | | | | | | | | | | | | | | | | | | | | | | | |
| | IncR | | | | | | | | | | | | | | | | | | | | | | | | |
| | IncFII(pKPX1) | | | | | | | | | | | | | | | | | | | | | | | | |
| | ColRNAI | | | | | | | | | | | | | | | | | | | | | | | | |
| | repB(R1701) | | | | | | | | | | | | | | | | | | | | | | | | |
| | IncP6 | | | | | | | | | | | | | | | | | | | | | | | | |
| | IncFIB(K)(pCAV1099-114) | | | | | | | | | | | | | | | | | | | | | | | | |
| | IncFIB(pNDM-Mar) | | | | | | | | | | | | | | | | | | | | | | | | |
| | IncFIB(pQil) | | | | | | | | | | | | | | | | | | | | | | | | |

**Supplementary Table 4**. Bacterial defence mechanisms against phage infection in the 24 carbapenemase-producing *Klebsiella pneumoniae* strains studied. Presence: grey, absence: white.

| ID strain | | 1589 | 1665 | 1674 | 1681 | 1890 | 1892 | 1898 | 1899 | 1907 | 1944 | 2100 | 5995 | 6106 | 1925 | 1954 | 1997 | 2096 | 2144 | 2173 | 3503 | 6022 | 2343 | 2465 | 2539 |
|---|---|---|---|---|---|---|---|---|---|---|---|---|---|---|---|---|---|---|---|---|---|---|---|---|---|
| MLST | | 15 | 15 | 15 | 15 | 15 | 15 | 15 | 15 | 15 | 15 | 15 | 17 | 147 | 147 | 147 | 147 | 147 | 147 | 392 | 147 | 307 | 147 | 449 | 1961 |
| BACTERIAL DEFENSE MECHANISMS | AbiD | | | | | | | | | | | | | | | | | | | | | | | | |
| | AbiE | | | | | | | | | | | | | | | | | | | | | | | | |
| | CAS | | | | | | | | | | | | | | | | | | | | | | | | |
| | CBASS | | | | | | | | | | | | | | | | | | | | | | | | |
| | Gao_Hhe | | | | | | | | | | | | | | | | | | | | | | | | |
| | Gao_Iet | | | | | | | | | | | | | | | | | | | | | | | | |
| | Gao_Tmn | | | | | | | | | | | | | | | | | | | | | | | | |
| | Hachiman | | | | | | | | | | | | | | | | | | | | | | | | |
| | MazEF | | | | | | | | | | | | | | | | | | | | | | | | |
| | Mok_Hok_Sok | | | | | | | | | | | | | | | | | | | | | | | | |
| | PD-T4-3 | | | | | | | | | | | | | | | | | | | | | | | | |
| | PD-T4-4 | | | | | | | | | | | | | | | | | | | | | | | | |
| | PD-T4-5 | | | | | | | | | | | | | | | | | | | | | | | | |
| | PD-T7-1 | | | | | | | | | | | | | | | | | | | | | | | | |
| | Retron | | | | | | | | | | | | | | | | | | | | | | | | |
| | RM | | | | | | | | | | | | | | | | | | | | | | | | |
| | Shango | | | | | | | | | | | | | | | | | | | | | | | | |
| | Gabija | | | | | | | | | | | | | | | | | | | | | | | | |
| | Rst_TIR-NLR | | | | | | | | | | | | | | | | | | | | | | | | |
| | Septu | | | | | | | | | | | | | | | | | | | | | | | | |
| | Pif | | | | | | | | | | | | | | | | | | | | | | | | |
| | Druantia | | | | | | | | | | | | | | | | | | | | | | | | |
| | Familia lamassu | | | | | | | | | | | | | | | | | | | | | | | | |
| | Rosmer TA | | | | | | | | | | | | | | | | | | | | | | | | |
| | SEFIR | | | | | | | | | | | | | | | | | | | | | | | | |
| | Zorya | | | | | | | | | | | | | | | | | | | | | | | | |
| | BREX | | | | | | | | | | | | | | | | | | | | | | | | |
| | PrrC | | | | | | | | | | | | | | | | | | | | | | | | |
| | SanaTA | | | | | | | | | | | | | | | | | | | | | | | | |

**Supplementary Table 5.** Depolymerase sequences detected in the genomes of the 6 Kpn bacteriophages selected for this study. The link to the sequence with the highest percentage of identity and the position of the sequence in the phage genome is provided.

| | | Percentage Identity (%) and Genomic Position per Phage (BLASTx, E-value <0.0001) | | | | | |
|---|---|---|---|---|---|---|---|
| ENTRY (UniProtKB reviewed) | Description | *Webervirus kpv33d1* | *Jiaodavirus kpv33d4* | *Jiaodavirus kpv33d6* | *Webervirus kpv33d7* | *Drulisvirus kpv33s1* | *Jiaodavirus kpvth1* |
| P0DTN7 | Depolymerase, capsule K2-specific | 82.5%, 23495 - 22872 | | | 95.1%, 23505 - 23137 | | |
| A0A9E7NFY6 | Tail/depolymerase | 72%, 9425 - 10078 | | | 94%, 9434 – 10087 | | |
| D1L2Y9 | Tail tubular protein A | | | | | 97.9%, 26132 - 26689 | |
| A0A068Q6B2 | Anchor protein | | | | | 68.2%, 36104 - 36883 | |

**Supplementary Table 6. Lytic activity of the selected phages against the 24 carbapenemase-producing *K. pneumoniae* strains of UTI origin in the Spot Test**. The logarithm of the highest phage dilution showing activity against each of the strains. Hypermucoviscosity phenotype (HMV+: Positive, NEG: negative) and MLST. The color represents the logarithm of phage dilution.

| ID | *Druslisvirus* kpv33s1 | *Webervirus* kpv33d1 | *Jiaodavirus* kpv33d4 | *Jiaodavirus* kpv33d6 | *Webervirus* kpv33d7 | *Jiaodavirus* kpvth1 | Nº PHAGE | HMV | MLST | |
|---|---|---|---|---|---|---|---|---|---|---|
| 1589 | 0 | 0 | 10 | 7 | 10 | 0 | 3 | HMV+ | 15 | |
| 1681 | 0 | 0 | 10 | 5 | 10 | 10 | 4 | NEG | 15 | |
| 1665 | 0 | 10 | 10 | 10 | 10 | 10 | 5 | HMV+ | 15 | |
| 1674 | 0 | 0 | 1 | 1 | 0 | 0 | 2 | NEG | 15 | |
| 1890 | 0 | 10 | 1 | 0 | 0 | 0 | 2 | NEG | 15 | |
| 1892 | 0 | 3 | 0 | 0 | 0 | 0 | 1 | HMV+ | 15 | |
| 1898 | 10 | 10 | 10 | 0 | 10 | 5 | 5 | HMV+ | 15 | |
| 1899 | 0 | 0 | 0 | 0 | 0 | 0 | 0 | NEG | 15 | |
| 1907 | 0 | 0 | 0 | 0 | 0 | 0 | 0 | HMV+ | 15 | |
| 1944 | 0 | 5 | 0 | 0 | 0 | 0 | 1 | HMV+ | 15 | |
| 2100 | 0 | 1 | 0 | 2 | 0 | 0 | 2 | NEG | 15 | |
| 1954 | 0 | 0 | 0 | 0 | 0 | 0 | 0 | HMV+ | 147 | |
| 1925 | 0 | 0 | 0 | 0 | 0 | 0 | 0 | HMV+ | 147 | |
| 1997 | 0 | 0 | 0 | 0 | 0 | 0 | 0 | HMV+ | 147 | |
| 2096 | 0 | 2 | 4 | 0 | 0 | 0 | 2 | HMV+ | 147 | |
| 2144 | 0 | 0 | 0 | 0 | 0 | 0 | 0 | HMV+ | 147 | |
| 2343 | 0 | 0 | 0 | 0 | 0 | 0 | 0 | HMV+ | 147 | |
| 6106 | 0 | 2 | 10 | 0 | 0 | 0 | 2 | NEG | 147 | |
| 3503 | 0 | 0 | 0 | 0 | 0 | 0 | 0 | HMV+ | 147 | |
| 2173 | 0 | 3 | 3 | 2 | 10 | 2 | 5 | HMV+ | 392 | |
| 2539 | 0 | 0 | 0 | 0 | 0 | 0 | 0 | NEG | 1961 | |
| 2465 | 10 | 2 | 1 | 0 | 0 | 0 | 3 | HMV+ | 449 | 8-10 |
| 5995 | 0 | 10 | 0 | 0 | 0 | 0 | 1 | NEG | 17 | 4-7 |
| 6022 | 1 | 1 | 2 | 2 | 1 | 1 | 6 | NEG | 307 | 1-3 |
| Nº LYSED STRAINS | 3 | 12 | 11 | 7 | 6 | 5 | | | | <1 |

**Supplementary Table 7**. **Lytic activity (FPU/mL) of the selected phages against the 24 carbapenemase-producing UTI *K. penumoniae* strains in the double agar plaque assay.** Hypermucoviscosity phenotype (HMV+: Positive, NEG: negative) and MLST. The color in the table represents the FPU/mL.

| PFU/ml | Druslisvirus kpv33s1 | Webervirus kpv33d1 | Jiaodavirus kpv33d4 | Jiaodavirus kpv33d6 | Webervirus kpv33d7 | Jiaodavirus kpvth1 | N°PHAGE | HMV | MLST |
|---|---|---|---|---|---|---|---|---|---|
| 1589 | 0 | 0 | 0 | 0 | 0 | 0 | 0 | HMV+ | 15 |
| 1681 | 0 | 0 | 0 | 0 | 0 | 0 | 0 | NEG | 15 |
| 1665 | 0 | 0 | 0 | 0 | 0 | 0 | 0 | HMV+ | 15 |
| 1674 | 0 | 0 | 0 | 0 | 0 | 0 | 0 | NEG | 15 |
| 1890 | 0 | 0 | 0 | 0 | 0 | 0 | 0 | NEG | 15 |
| 1892 | 0 | 0 | 0 | 0 | 0 | 0 | 0 | HMV+ | 15 |
| 1898 | 0 | 0 | 0 | 0 | 0 | 0 | 0 | HMV+ | 15 |
| 1899 | 0 | 0 | 0 | 0 | 0 | 0 | 0 | NEG | 15 |
| 1907 | 0 | 0 | 0 | 0 | 0 | 0 | 0 | HMV+ | 15 |
| 1944 | 0 | 0 | 0 | 0 | 0 | 0 | 0 | HMV+ | 15 |
| 2100 | 0 | 0 | 0 | 0 | 0 | 0 | 0 | NEG | 15 |
| 1954 | 0 | 0 | 0 | 0 | 0 | 0 | 0 | HMV+ | 147 |
| 1925 | 0 | 0 | 0 | 0 | 0 | 0 | 0 | HMV+ | 147 |
| 1997 | 0 | 0 | 0 | 0 | 0 | 0 | 0 | HMV+ | 147 |
| 2096 | 0 | 2,11E+04 | 0 | 0 | 0 | 4,70E+03 | 2 | HMV+ | 147 |
| 2144 | 0 | 0 | 0 | 0 | 0 | 0 | 0 | HMV+ | 147 |
| 2343 | 0 | 0 | 0 | 0 | 0 | 0 | 0 | HMV+ | 147 |
| 6106 | 0 | 0 | 3,80E+04 | 0 | 0 | 0 | 1 | NEG | 147 |
| 3503 | 0 | 0 | 0 | 0 | 0 | 0 | 0 | HMV+ | 147 |
| 2173 | 0 | 2,9E+15 | 3,8E+13 | 5,1E+15 | 3,0E+15 | 4,4E+15 | 5 | HMV+ | 392 |
| 2539 | 0 | 0 | 0 | 0 | 0 | 0 | 0 | NEG | 1961 |
| 2465 | 0 | 0 | 0 | 0 | 0 | 0 | 0 | HMV+ | 449 |
| 5995 | 0 | 7,1E+05 | 0 | 0 | 0 | 2,0E+02 | 2 | NEG | 17 |
| 6022 | 9,6E+03 | 0 | 6,4E+03 | 0 | 2,0E+05 | 0 | 3 | NEG | 307 |
| **N° LYSED STRAINS** | **1** | **3** | **3** | **1** | **2** | **3** | | | |
| **KLEB-33 (PFU/ml)** | 3,0E+12 | 1,8E+15 | 5,0E+12 | 1,4E+14 | 1,1E+16 | 1,2E+16 | | | |

| |
|---|
| 10E+11-10E+15 |
| 10E+06-10E+10 |
| 10-10E+5 |
| <10 |

## Webervirus

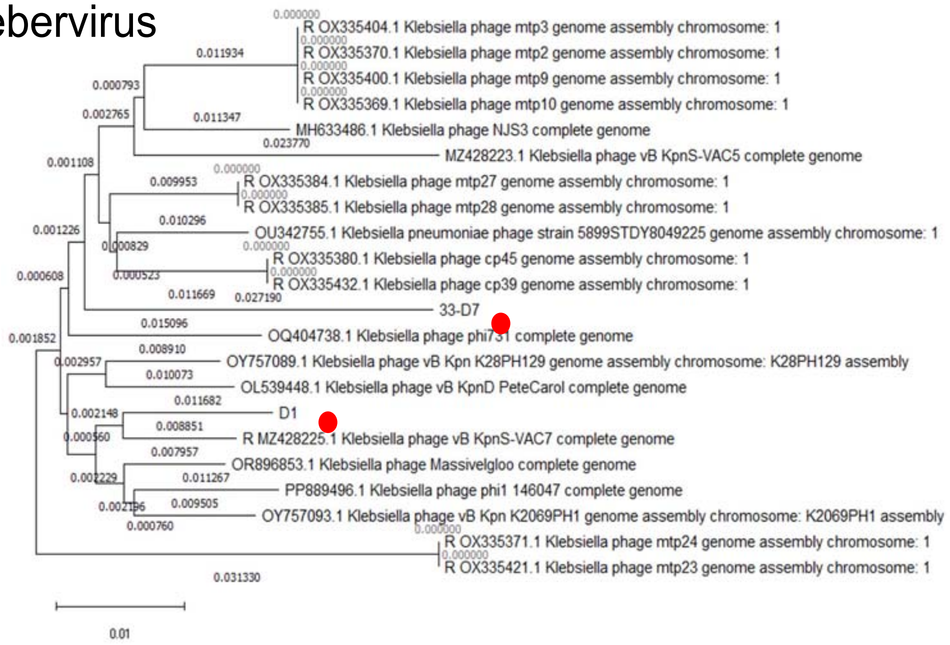


## jiaodavirus

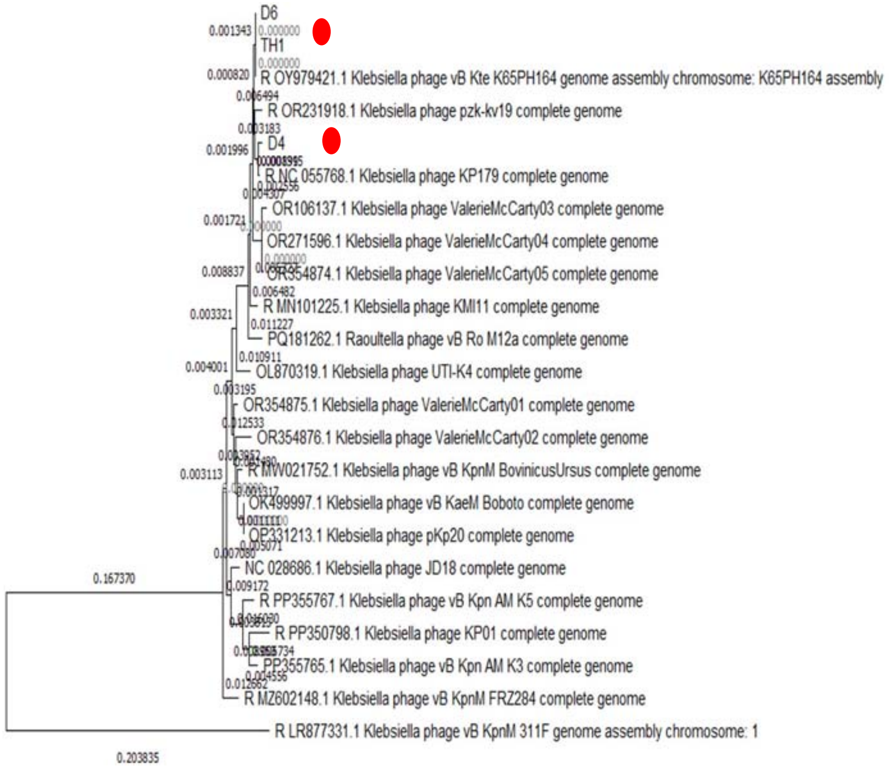

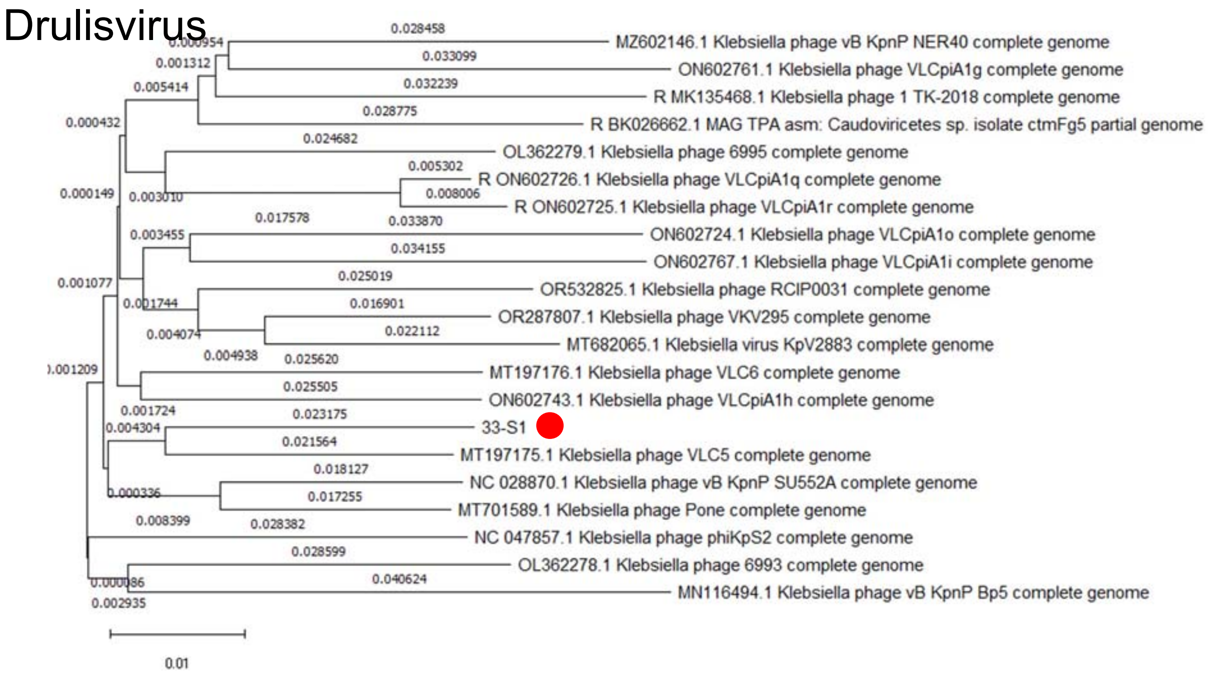


**Supplementary Figure 1**. Taxonomic position of the 6 *Klebsiella pneumoniae* bacteriophages selected for this study.
